# Supplementary material for: A nitric-oxide driven chemotactic nanomotor for enhanced immunotherapy of glioblastoma
Source: Nat Commun. 2023 Feb 20;14:941. doi: 10.1038/s41467-022-35709-0 (PMC9941476; doi:10.1038/s41467-022-35709-0)
Supplement: Supplementary file 1 — Supplementary Information [file 41467_2022_35709_MOESM1_ESM.pdf]

Supplementary Information

A nitric-oxide driven chemotactic nanomotor for enhanced immunotherapy of glioblastoma

Huan Chen[1,†], Ting Li[1,†], Zhiyong Liu[1,†], Shuwan Tang[1], Jintao Tong[2], Yingfang Tao[1], Zinan Zhao[1], Nan Li[1], Chun Mao[1,*], Jian Shen[1], Mimi Wan[1,*]

[1]National and Local Joint Engineering Research Center of Biomedical Functional Materials, School of Chemistry and Materials Science, Nanjing Normal University, Nanjing 210023, China.

[2] College of Chemistry and Molecular Engineering, Peking University, Beijing 100871, China.

* Corresponding author: Chun Mao, Mimi Wan

E-mail: C. M. (maochun@njnu.edu.cn); M. M. W. (wanmimi@njnu.edu.cn).

[†]These authors contributed equally.

Supplementary figures and tables

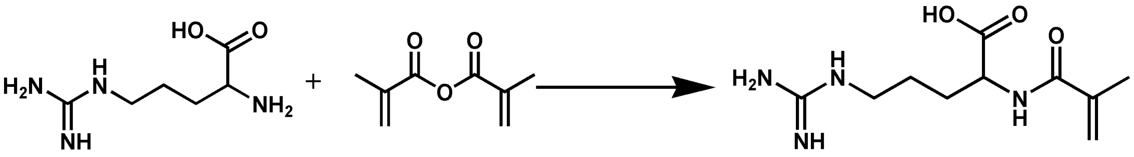

Supplementary Fig. 1 Synthesis process of arginine-methacryloylamide (Arg-Me).

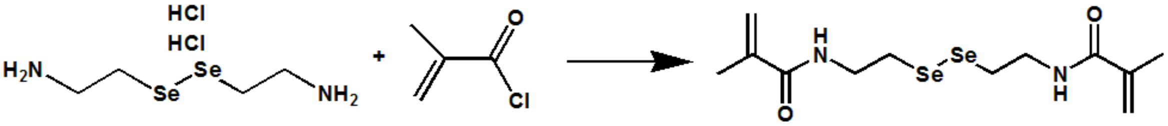

Supplementary Fig. 2 Synthesis process of diselenide cross-linker.

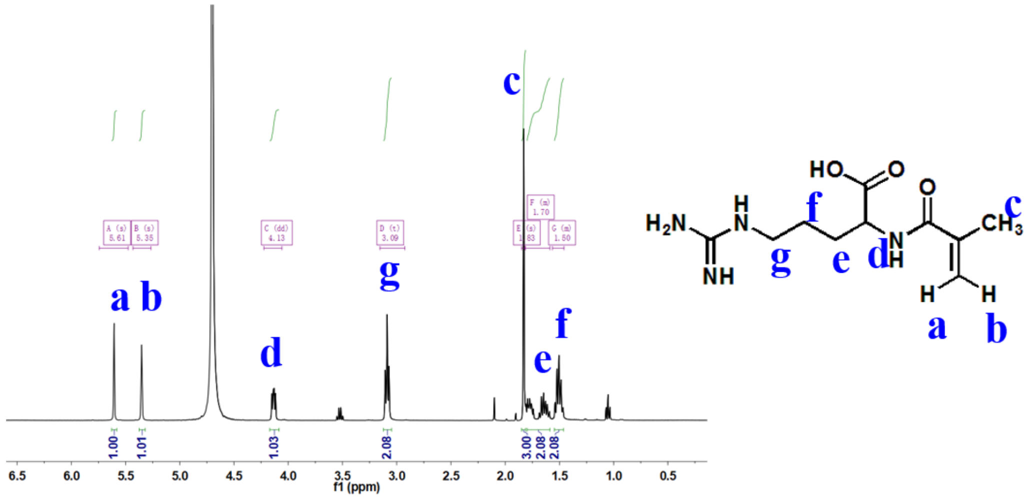

Supplementary Fig. 3 ^{1}H NMR spectrum of Arg-Me (D_2O, 400 MHz). ^{1}H NMR (400 MHz, D_2O) δ 5.61 (s, 1H), 5.35 (s, 1H), 4.13 (dd, J = 8.0, 5.1 Hz, 1H), 3.09 (t, J = 6.8 Hz, 2H), 1.83 (s, 3H), 1.81 – 1.59 (m, 2H), 1.56 – 1.46 (m, 2H), indicating the successful synthesis of Arg-Me.

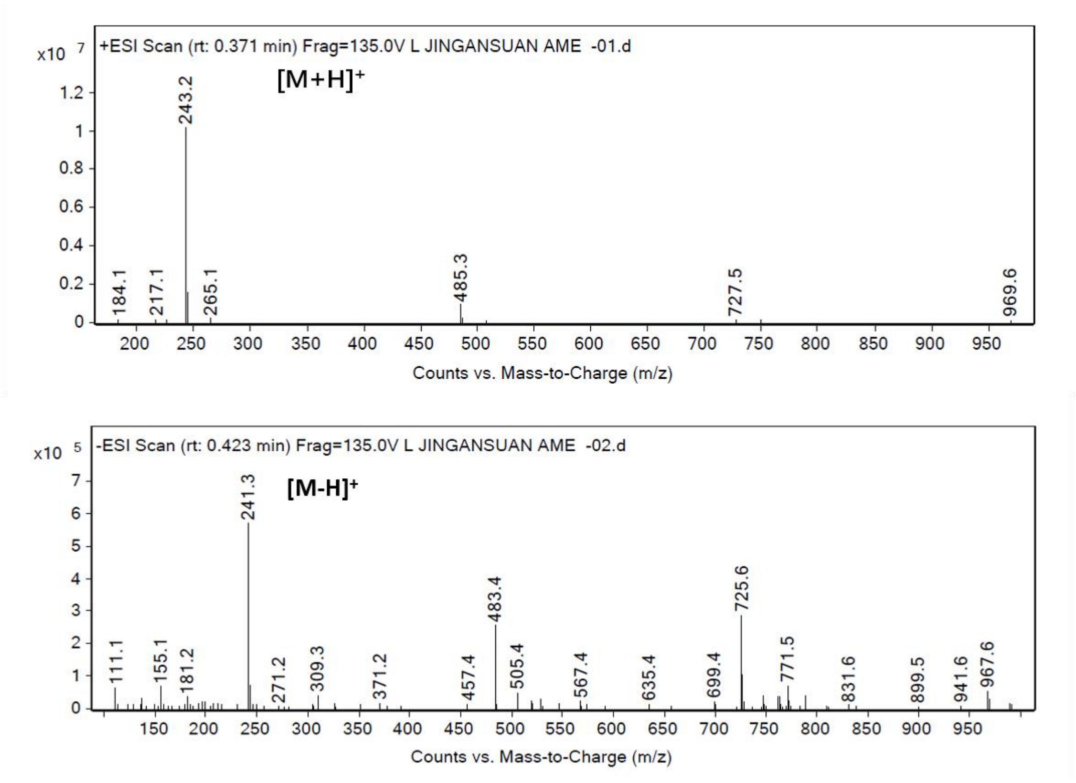

Supplementary Fig. 4 The mass spectrometry of Arg-Me.

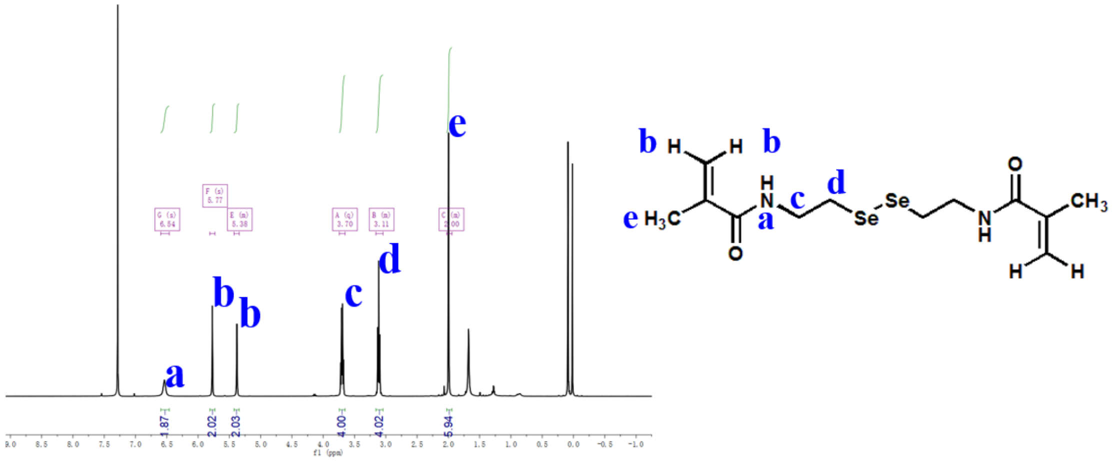

Supplementary Fig. 5 ^{1}H NMR spectrum of diselenide cross-linker (d-$CDCl_3$, 400 MHz). ^{1}H NMR (400 MHz, $CDCl_3$) δ 6.54 (s, 2H), 5.77 (s, 2H), 5.43-5.34 (m, 2H), 3.70 (q, J = 6.5 Hz, 4H), 3.16-3.04 (m, 4H), 2.03-1.94 (m, 6H), 1.68 (s, 4H), indicating the successful synthesis of diselenide cross-linker.

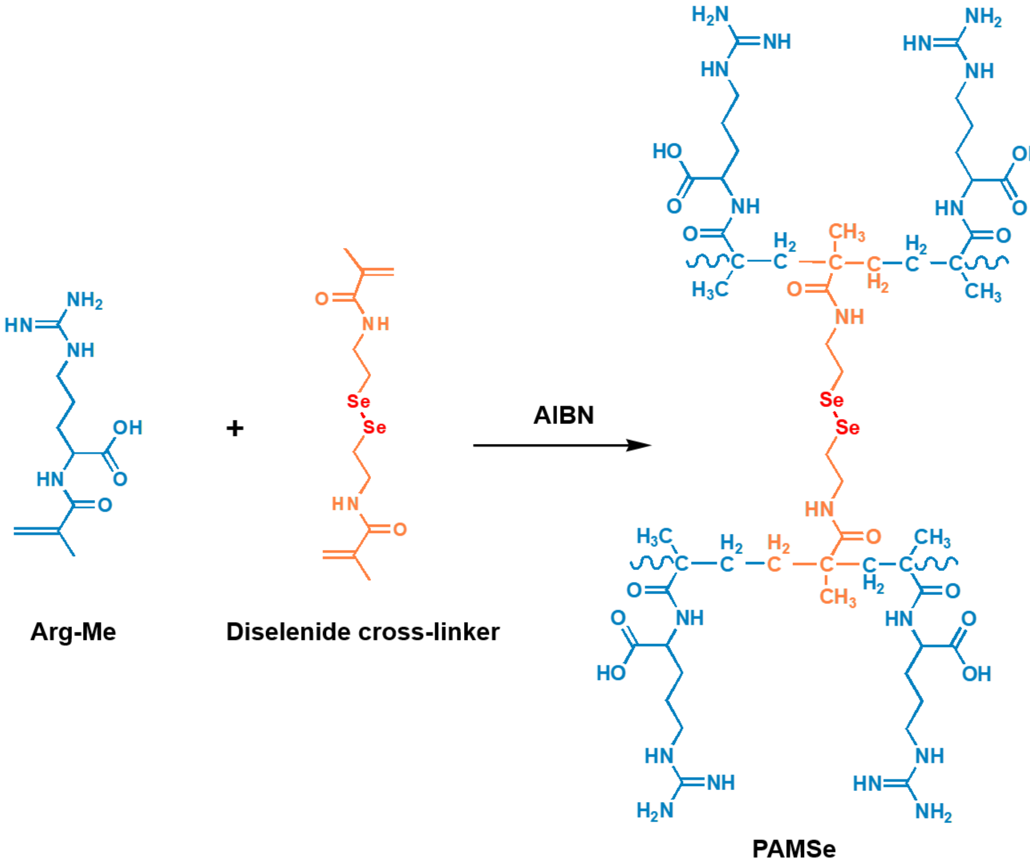

Supplementary Fig. 6 Synthesis route of PAMSe nanomotors.

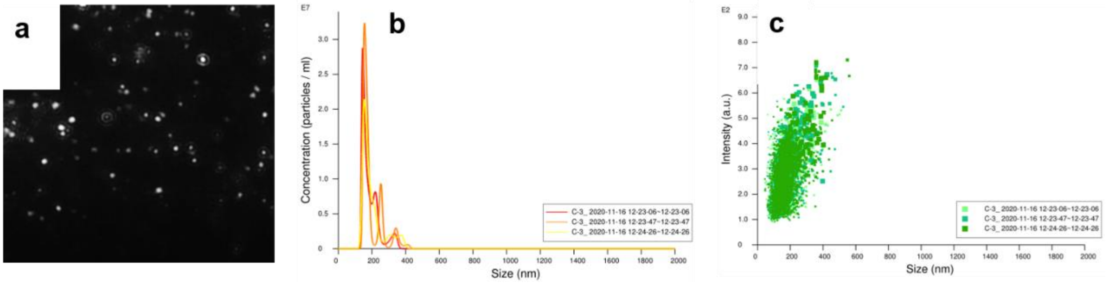

Supplementary Fig. 7 Size distribution from nanoparticle tracking analysis and DLS measurements of mixtures of monodisperse PAMSe nanomotors. **a** The corresponding NTA video frame. **b** Averaged nanoparticle concentration/size, and (**c**) Size graph for experiment. The concentration of nanomotor is about 1.42e+009 +/- 3.12e+007 particles mL^{-1} in 200 g mL^{-1} solution (n=3 independent samples).

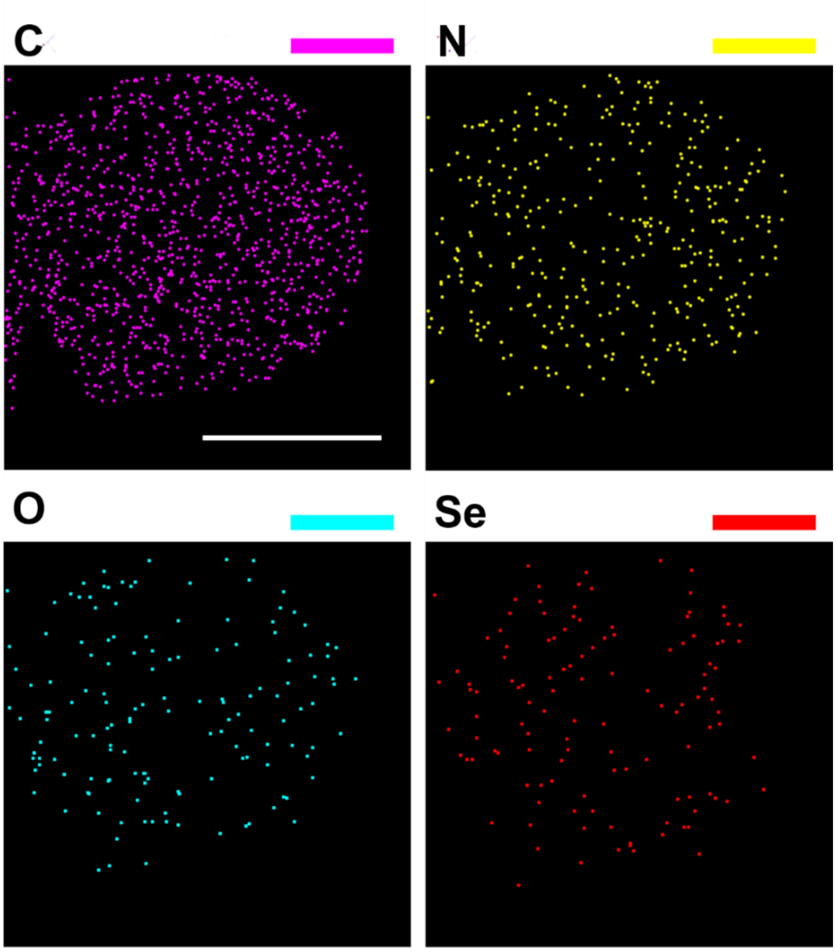

Supplementary Fig. 8 TEM-assisted element mapping images of PAMSe nanomotors (n=3 independent experiments, Scale bar: 100 nm).

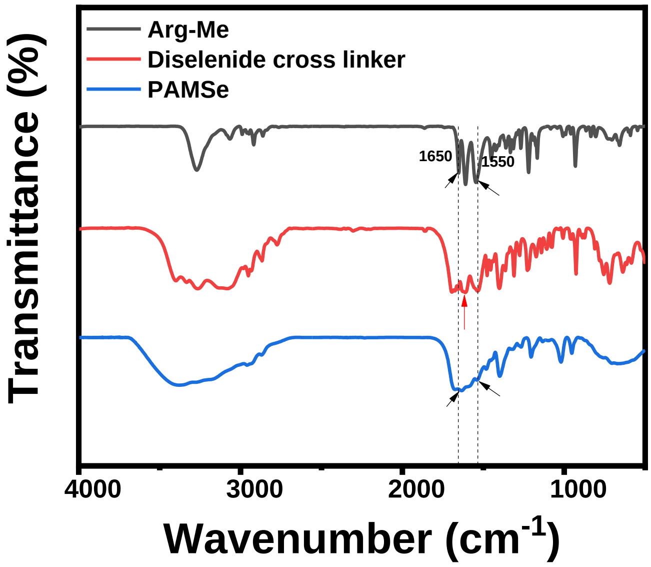

Supplementary Fig. 9 FTIR spectra of Arg-Me, diselenide cross-linker, and PAMSe (n=3 independent samples).

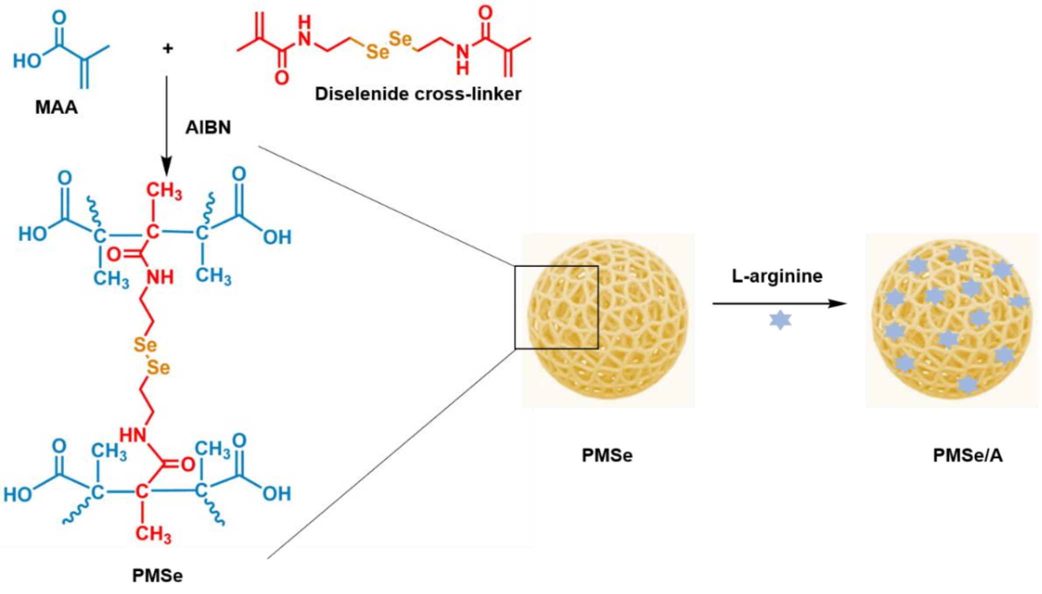

Supplementary Fig. 10 Synthesis route of post-loaded PMSe/A nanomotors.

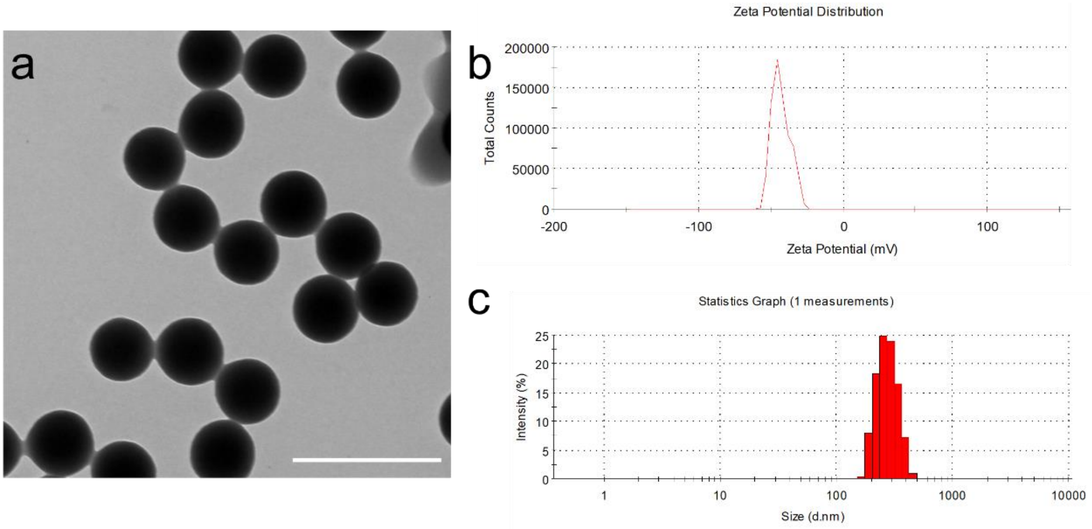

Supplementary Fig. 11 Characterization of the post-loaded PMSe/A nanomotors. a TEM image (scale bar: 500 nm), (**b**) Zeta potential, and (**c**) particle size distributions of PMSe/A (n=3 independent samples).

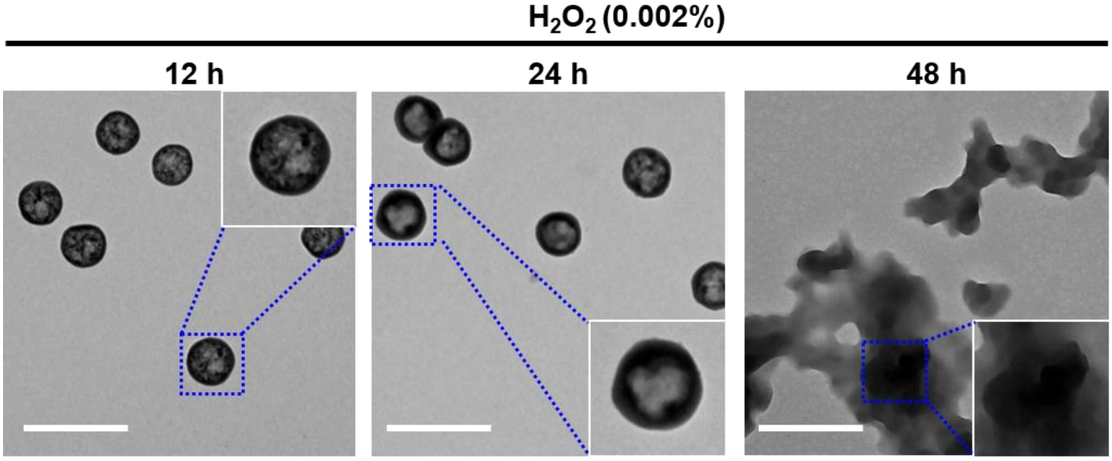

Supplementary Fig. 12 TEM images of PAMSe nanomotors after being incubated in simulated ROS environment (0.002% H_2O_2) for 12, 24 and 48 h (Scale bar: 500 nm). Data shown in three independent samples.

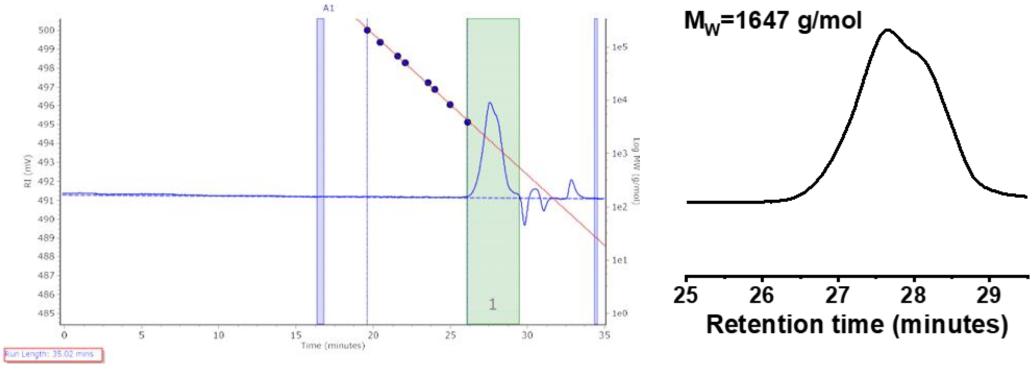

Supplementary Fig. 13 GPC trace of the polymer chain of PAMSe nanomotors degraded in H_2O_2 (0.002%) solution for 48 h (n=3 independent samples).

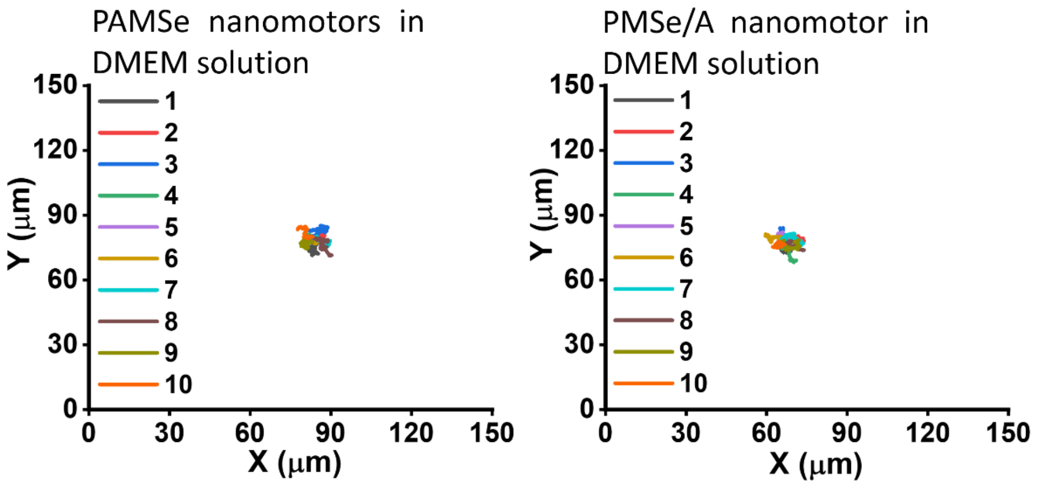

Supplementary Fig. 14 Trajectory of PAMSe nanomotors and PMSe/A nanomotors in DMEM solution (Supplementary Movie 3, 10 s,). Samples in a representative experiment, n = 10.

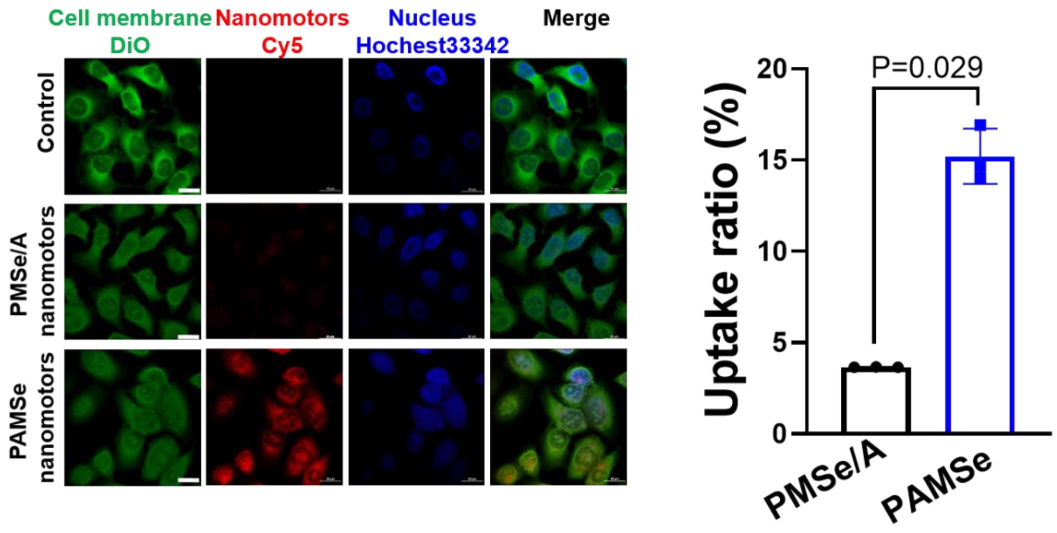

Supplementary Fig. 15 Representative CLSM images and uptake ratio of cancer cells after uptaking the cy5- PMSe/A or cy5-PAMSe nanomotors (Green, cell membranes stained with DiO; red, nanomotors were fluorescently labeled with cy5; blue, cell nuclei stained with hochest33342; Scale bar: 20 μm). Data shown as the mean ± s.d. (n=3 biologically independent experiments per group). Statistical significance was assessed by independent samples t-test (unpaired two sample t-test). Source data are provided as a Source Data file.

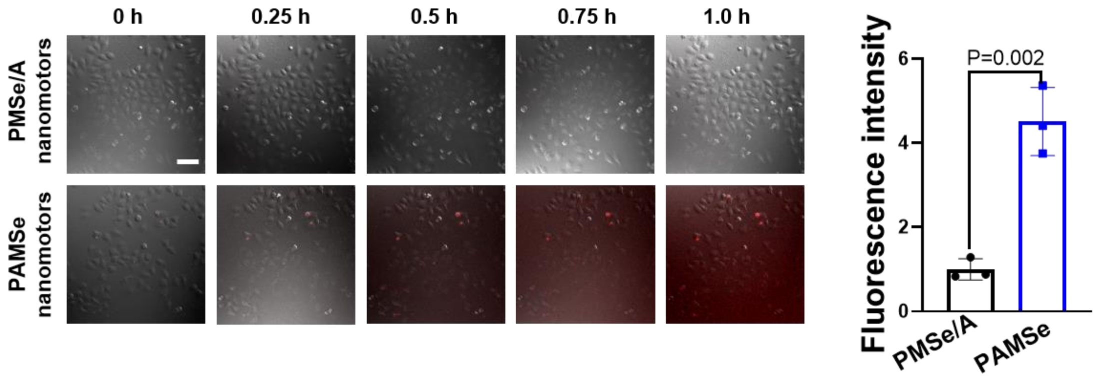

Supplementary Fig. 16 Real-time live-cell images (Supplementary Movie 4) of cancer cell treatment with PMSe/A or PAMSe nanomotors and the corresponding fluorescence intensity (F. I.) of nanomotors at t=1 h. (Bright, cells; red, nanomotors labeled with cy5; scale bar: 100 μm). Data shown as the mean ± s.d. (n=3 biologically independent experiments per group). Statistical significance was assessed by independent samples t-test (unpaired two sample t-test). Source data are provided as a Source Data file.

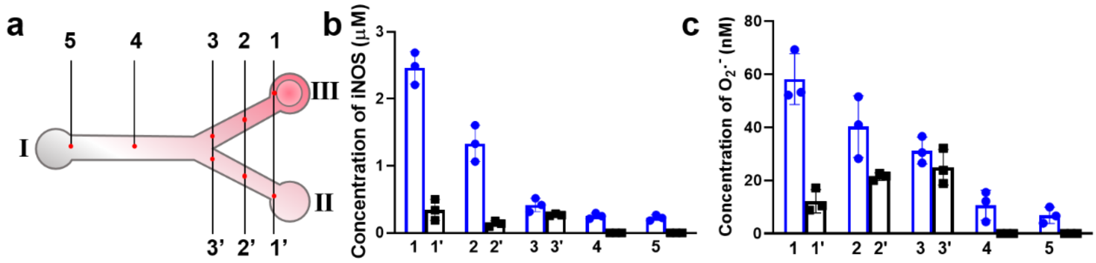

Supplementary Fig. 17 The concentration gradient of iNOS and ROS ($O_2{\cdot}^-$) in the Y-shaped channel. **a** The cell lysate of bEnd.3 or GL261 cells at initial density of 5×10^6 cells mL^{-1} was mixed with the equal volume of agarose solution in reservoir (II) and (III) respectively to form the network gels. After the agarose had solidified and stabilized for 30 min, the concentrations of (**b**) iNOS and (**c**) $O_2{\cdot}^-$ were detected at the corresponding positions, respectively (n=3). Data shown as the mean ± s.d. (n=3 biologically independent experiments per group). Source data are provided as a Source Data file.

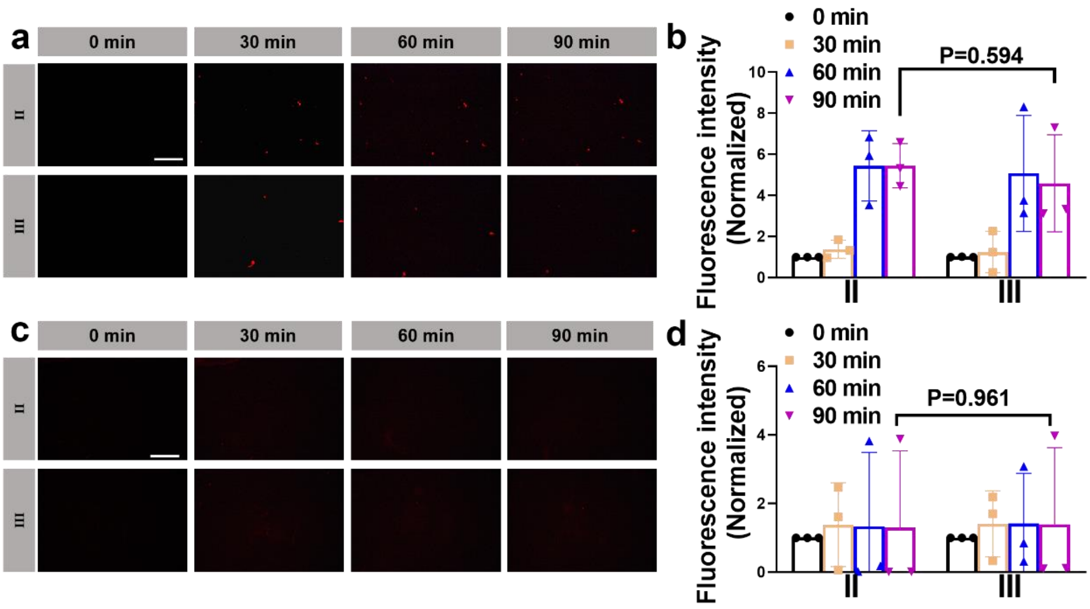

Supplementary Fig. 18 Chemotactic dynamics of samples upon exposure to chemokine gradient. a Representative CLSM images and (**b**) their fluorescence quantification taken at intervals after loading non-nanomotors PMSe into the Y model at chamber (I) and inoculating cancer cells (GL-261) into one side of chamber (III) while chamber (II) was inoculated with brain endothelial cells (bEnd.3) (n=3). **c** The nanomotors PAMSe was loaded into the Y model at chamber (I), and both channels of the Y model were inoculated with brain endothelial cells, and confocal pictures were taken at intervals and (**d**) corresponding fluorescence quantification (Scale bar: 400 μm). Data shown as the mean ± s.d. (n=3 independent experiments per group). Statistical significance was assessed by independent samples t-test (unpaired two sample t-test). Source data are provided as a Source Data file.

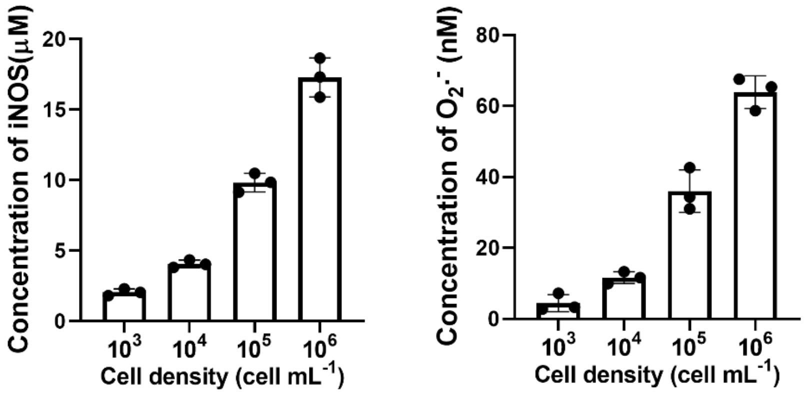

Supplementary Fig. 19 The intracellular concentration of iNOS and $O_2{\cdot}^-$ in GL261 cancer cells with different cell densities. Data shown as the mean ± s.d. (n=3 independent experiments per group). Source data are provided as a Source Data file.

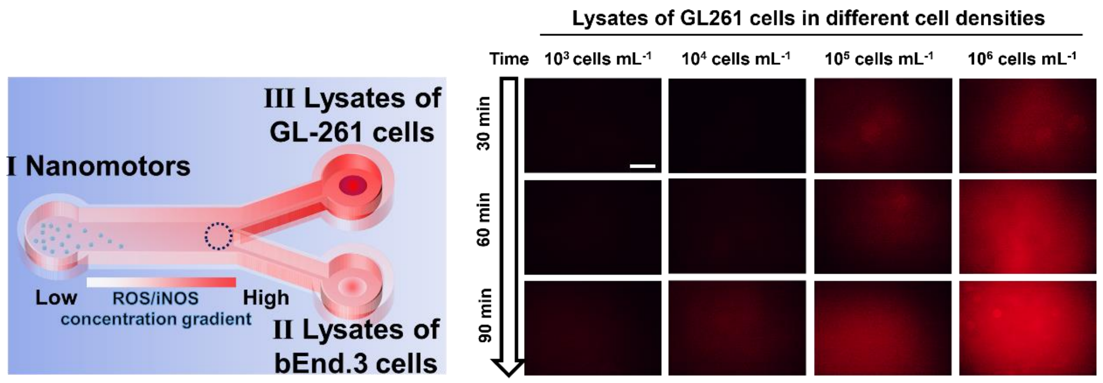

Supplementary Fig. 20 Diagram of Y-shaped channel and representative fluorescence images of PAMSe nanomotors in reservoir (III) designated position of Y-shaped channel at 30 min, 60 min and 90 min. Among them, the agarose gels in reservoir (III) and (II) containing equal volume of agarose solution and GL261or bEnd.3 cells lysate with different cell densities (10^3, 10^4, 10^5 and 10^6 cells mL^{-1}) (Scale bar: 400 μm, n=3 independent experiments per group).

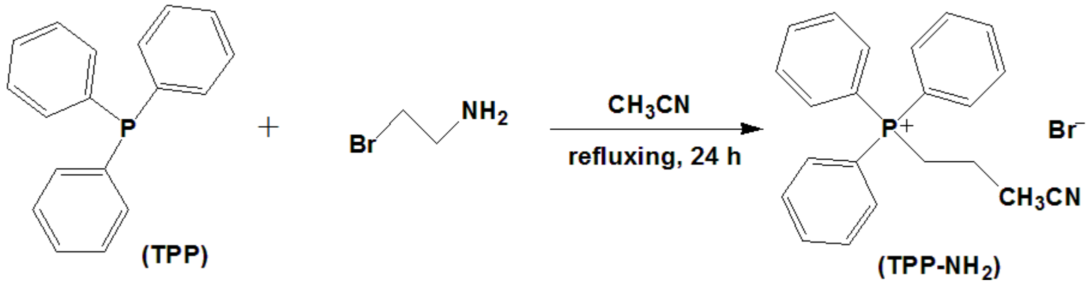

Supplementary Fig. 21 Synthesis of amino functionalized triphenylphosphine (TPP-NH_2).

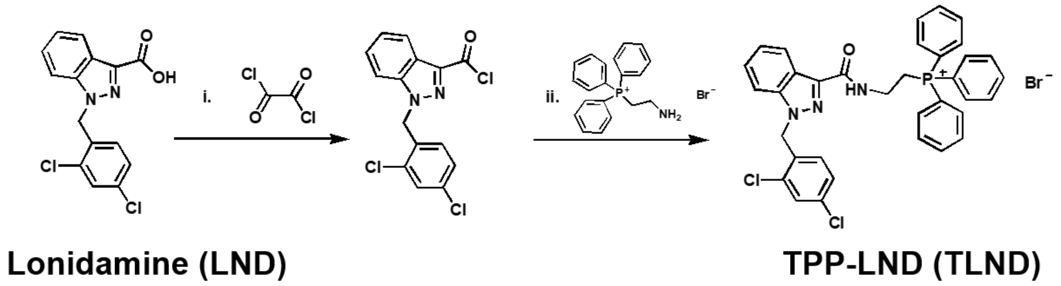

Supplementary Fig. 22 Diagram of the reaction mechanism of synthesis TLND.

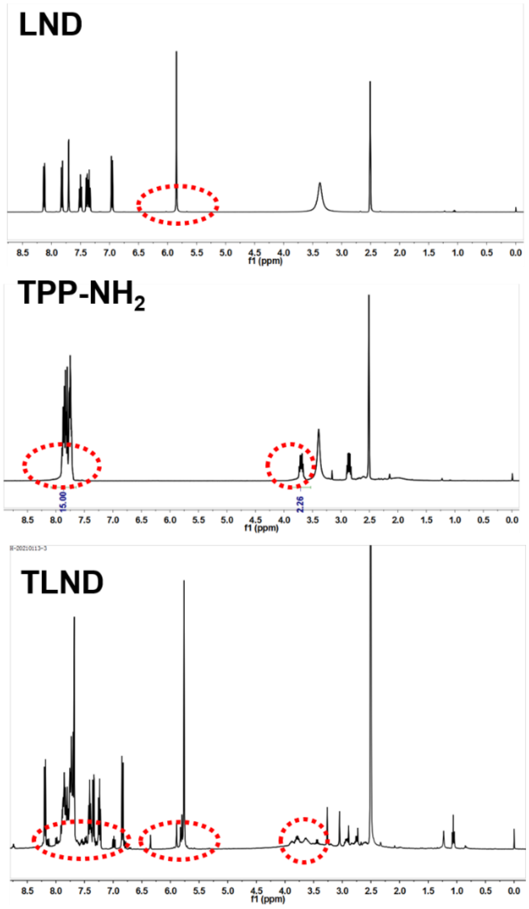

Supplementary Fig. 23 ^{1}H NMR spectrum of LND, TPP-NH_2 and TLND (d-DMSO, 400 MHz).

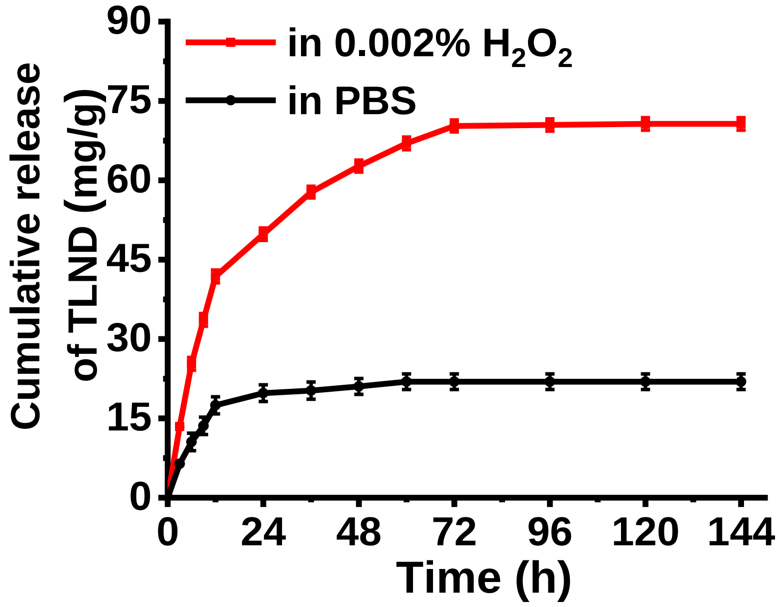

Supplementary Fig. 24 Cumulative drug release profiles of PAMSe/TLND in PBS solution and PBS solution containing 0.002% H_2O_2. Data shown as the mean ± s.d. (n=3 samples per group). Source data are provided as a Source Data file.

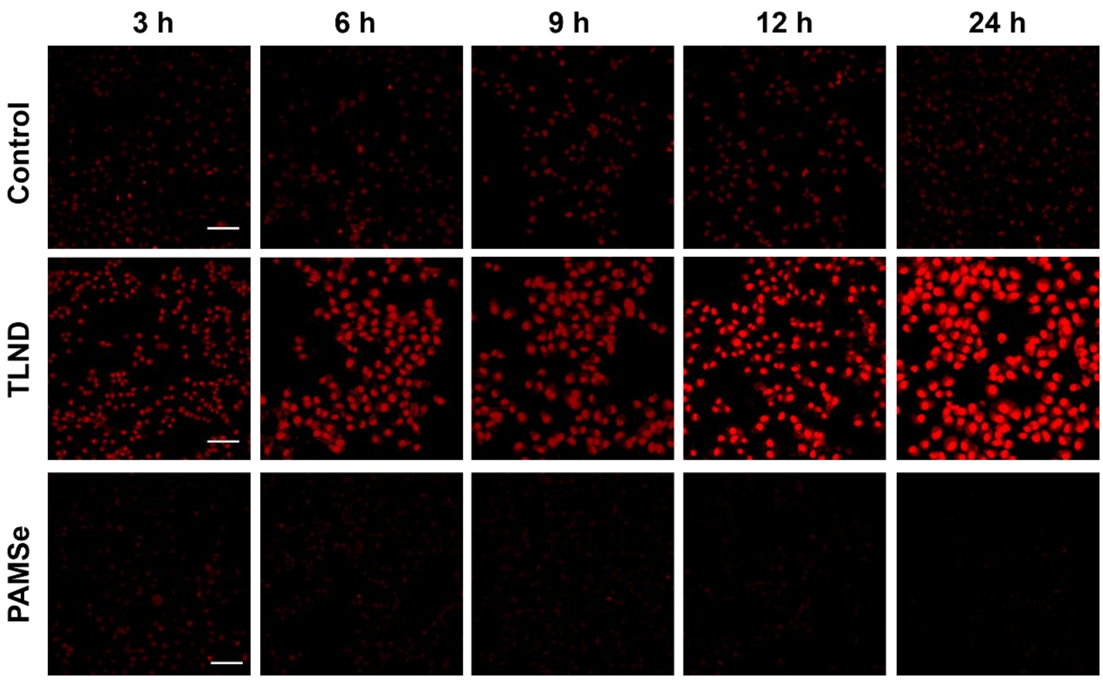

Supplementary Fig. 25 Representative CLSM images of intracellular ROS (labeled with the ROS fluorescent probe, DCFH-DA) in cancer cells after TLND and PAMSe treatment for 24 h (n=3 samples, Scale bar: 100 μm).

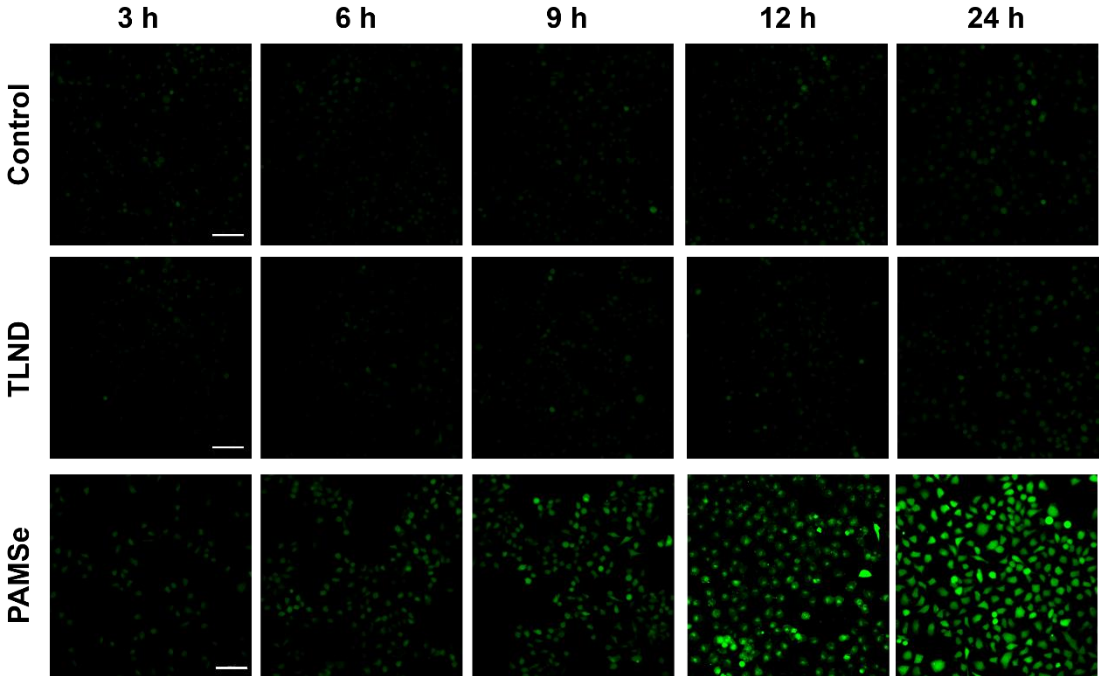

Supplementary Fig. 26 Representative CLSM images of intracellular NO (labeled with the NO fluorescent probe, DAF-FM DA) in cancer cells after TLND and PAMSe treatment for 24 h (n=3 samples, Scale bar: 100 μm).

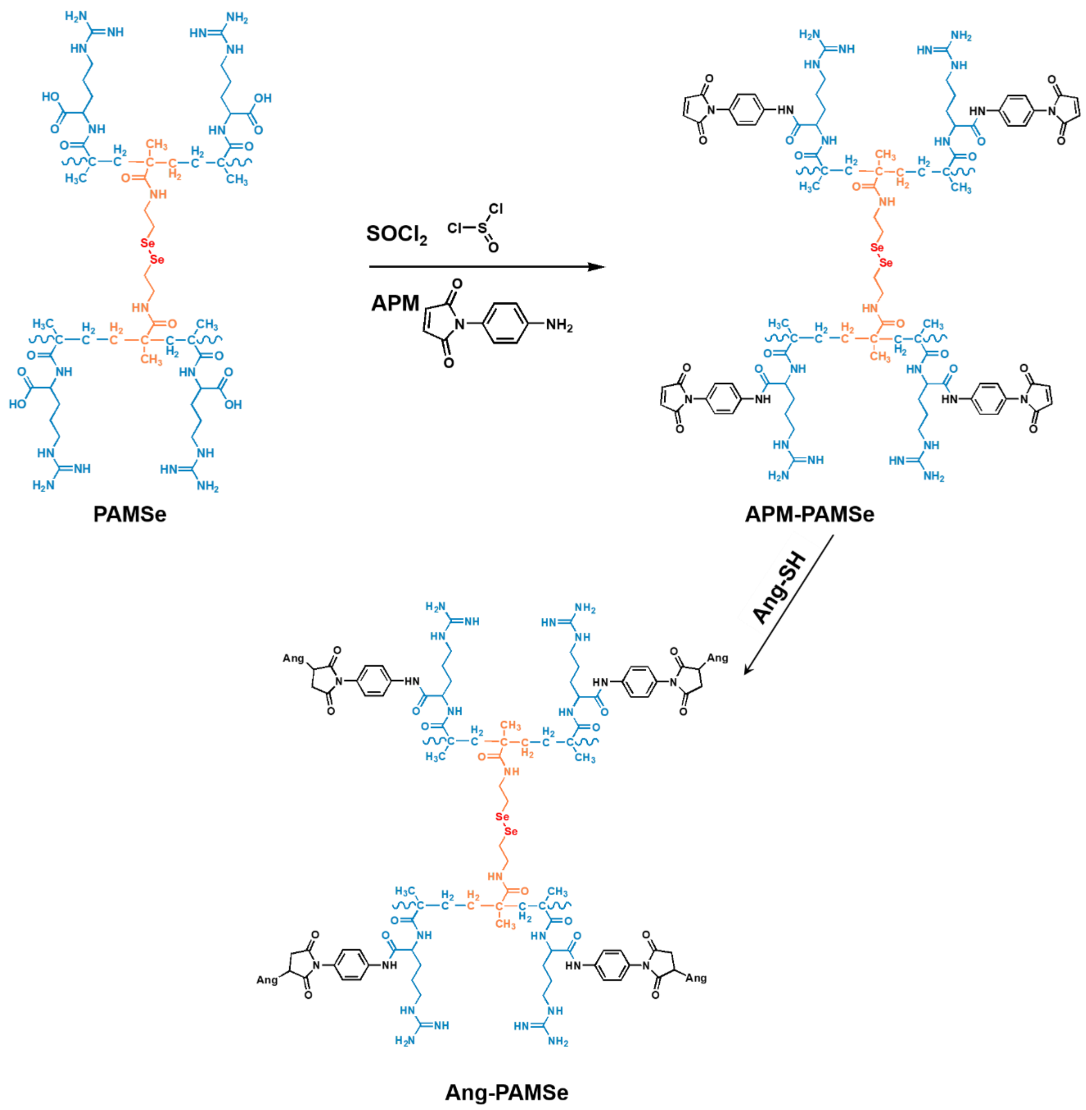

Supplementary Fig. 27 The synthetic routes of Ang-PAMSe.

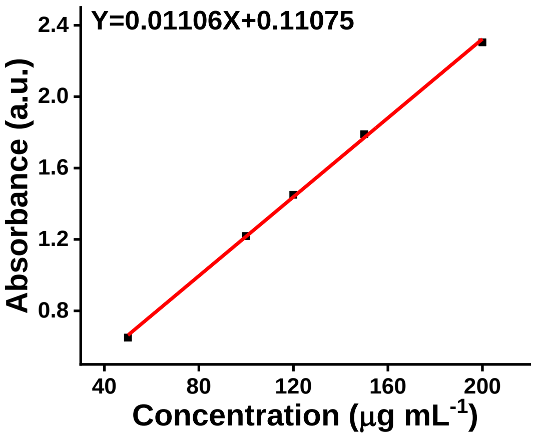

Supplementary Fig. 28 Standard concentration curve of Angiopep-2 (UV absorption wavelength at 214 nm).

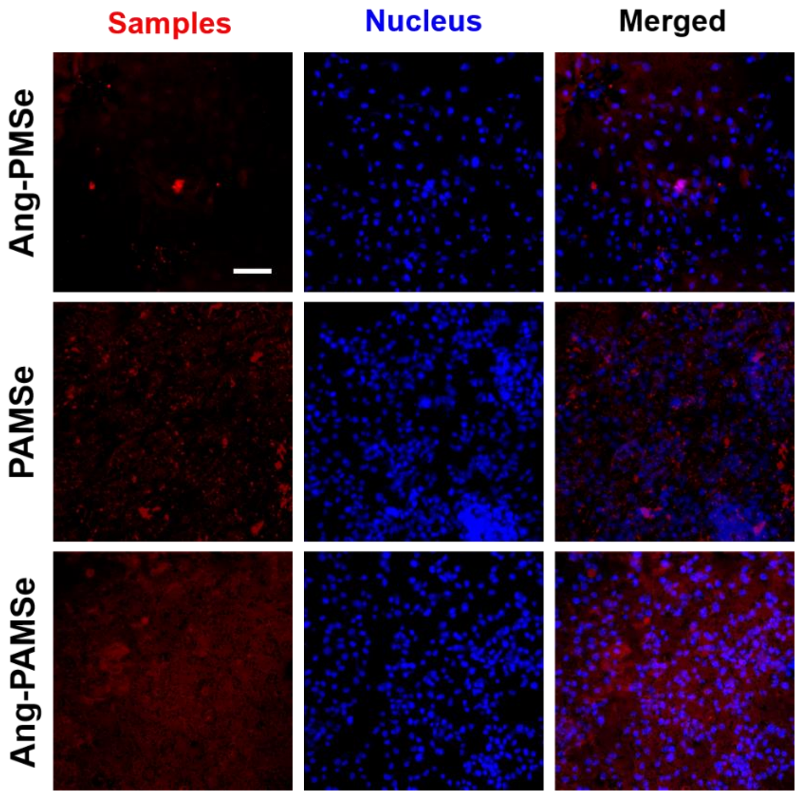

Supplementary Fig. 29 Representative CLSM images corresponding to schematic 4a of the lower chamber after adding samples to the upper chamber of the transwell and incubating for 24 h (corresponding to Fig. 4a) (Red, samples were fluorescently labeled with cy5; blue, cell nuclei stained with hochest33342; Scale bar: 100 μm, n=3 samples).

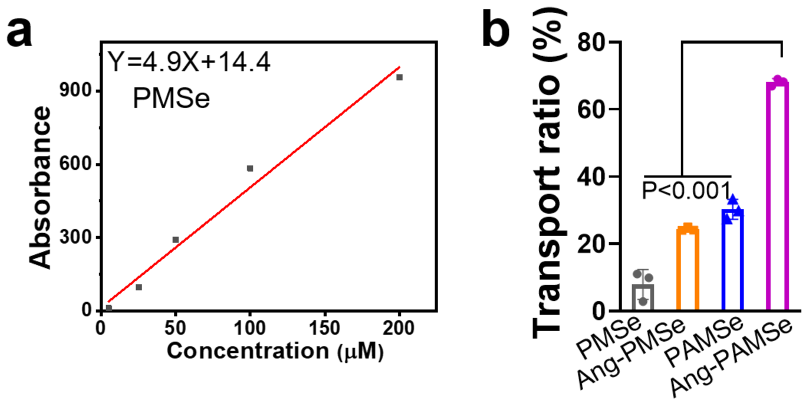

Supplementary Fig. 30 BBB transport ratio. **a** Concentration and fluorescence-worthy standard curve of PMSe. **b** BBB transport ratio of different sample. Data shown as the mean ± s.d. (n=3 independent experiments per group). Statistical significance was assessed by one-way ANOVA with post hoc LSD tests. Source data and exact p values are provided in the Source data file.

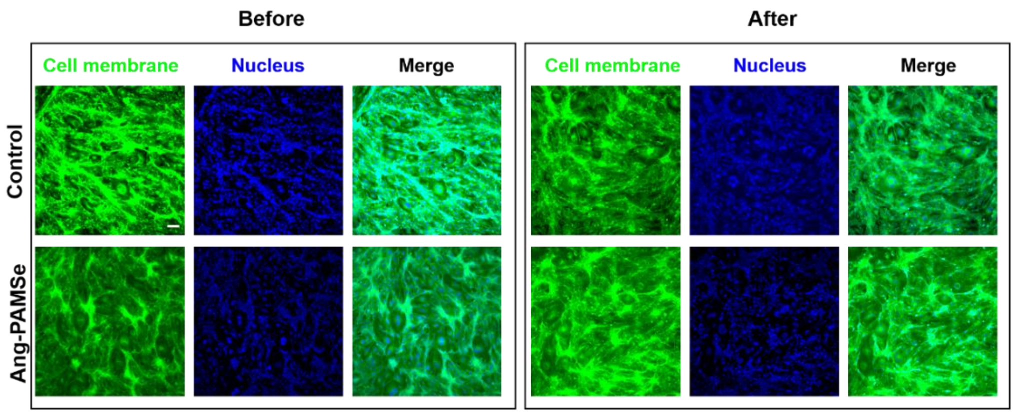

Supplementary Fig. 31 CLSM plots of the BBB in vitro (before) and after the addition of fresh medium (control) and Ang-PAMSe nanomotors (200 μg mL^{-1}) and incubation for 24 h (after). (Green, cell membranes stained with DiO; Blue, cell nuclei stained with DAPI; Scale bar: 100 μm, n=3 samples)

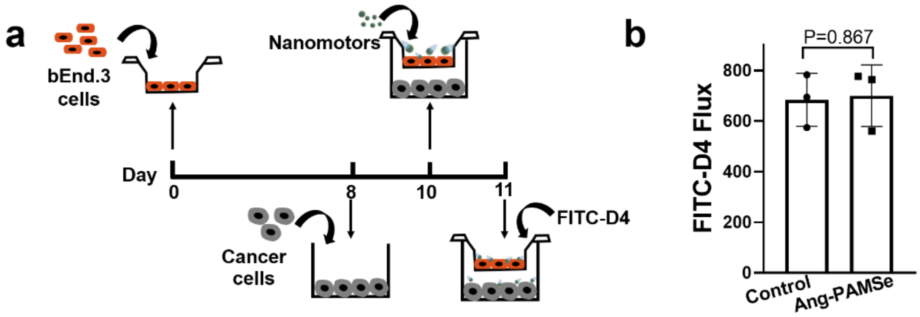

Supplementary Fig. 32 a Schematic representation of BBB integrity assessment using FD-4. **b** Paracellular permeability of FD-4 after 24 h treatment with Ang-PAMSe nanomotor. Data shown as the mean ± s.d. (n=3 independent experiments per group). Statistical significance was assessed by independent samples t-test (unpaired two sample t-test). Source data are provided as a Source Data file.

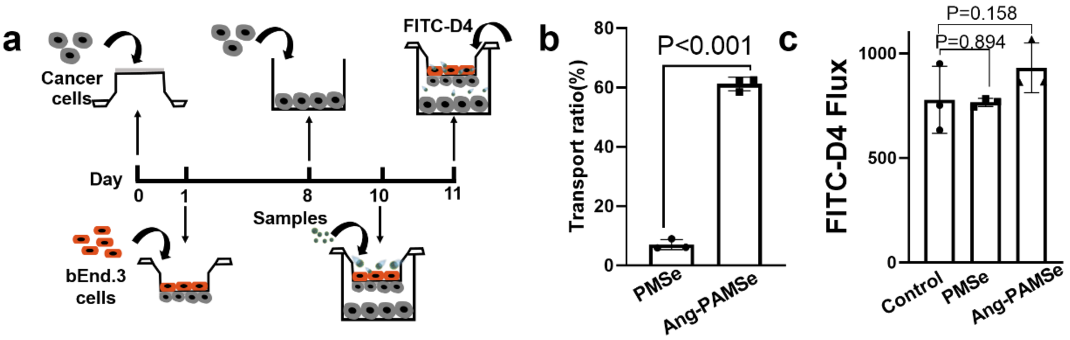

Supplementary Fig. 33 a Schematic diagram of in vitro multilayer symbiotic BBB model preparation. **b** Cellular transport rates of PMSe and Ang-PAMSe in a multilayer symbiotic BBB model. Statistical significance was assessed by independent samples t-test (unpaired two sample t-test). **c** Paracellular permeability of FD-4 after 24 h treatment with PMSe and Ang-PAMSe (The control group was fresh cell culture medium). Statistical significance was assessed by one-way ANOVA with post hoc LSD tests. Data shown as the mean ± s.d. (n=3 independent experiments per group). Source data and exact p values are provided in the Source data file.

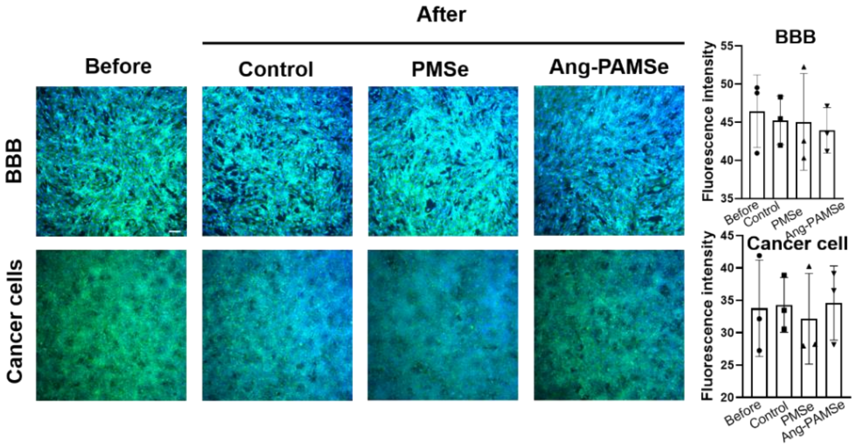

Supplementary Fig. 34 CLSM plots and fluorescence quantification of the upper co-cultured BBB and cancer cell layers in an in vitro multilayer symbiotic BBB model after adding fresh medium (control), PMSe and Ang-PAMSe nanomotors (200 μg mL^{-1}) and incubating for 24 h (after) (Green, cell membranes stained with DiO; Blue, cell nuclei stained with DAPI; Scale bar: 100 μm). Data shown as the mean ± s.d. (n=3 independent experiments per group). Source data are provided as a Source Data file.

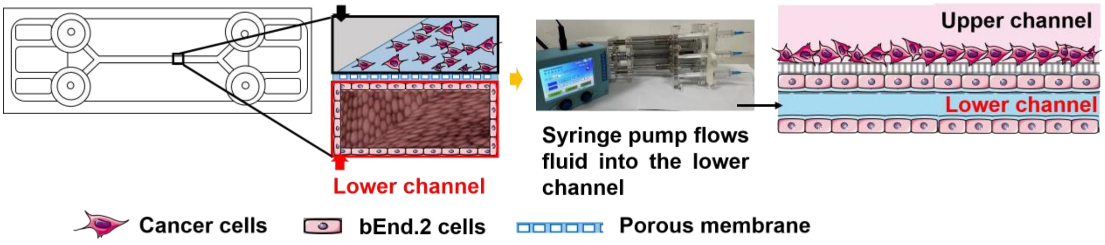

Supplementary Fig. 35 Schematic diagram of constructing an in vitro BBB model under dynamic condition. Photographs of the microfluidic chip (left), schematic diagram inside the channel (middle), with bEnd.2 cells cultured on all surfaces of the lower channel and glioblastoma cells (GL261) inoculated on the porous membrane surface of the upper channel. Schematic diagram of the passage of fluid through the syringe pump (Right).

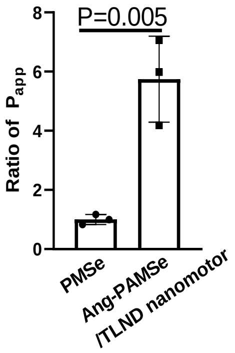

Supplementary Fig. 36 Transcytosis of PMSe and Ang-PAMSe nanomotors, measured by quantifying the ratio of P_{app} of Ang-PAMSe nanomotors versus PMSe passive nanoparticles in BBB chips. Data shown as the mean ± s.d. (n=3 independent experiments per group). Statistical significance was assessed by independent samples t-test (unpaired two sample t-test). Source data are provided as a Source Data file.

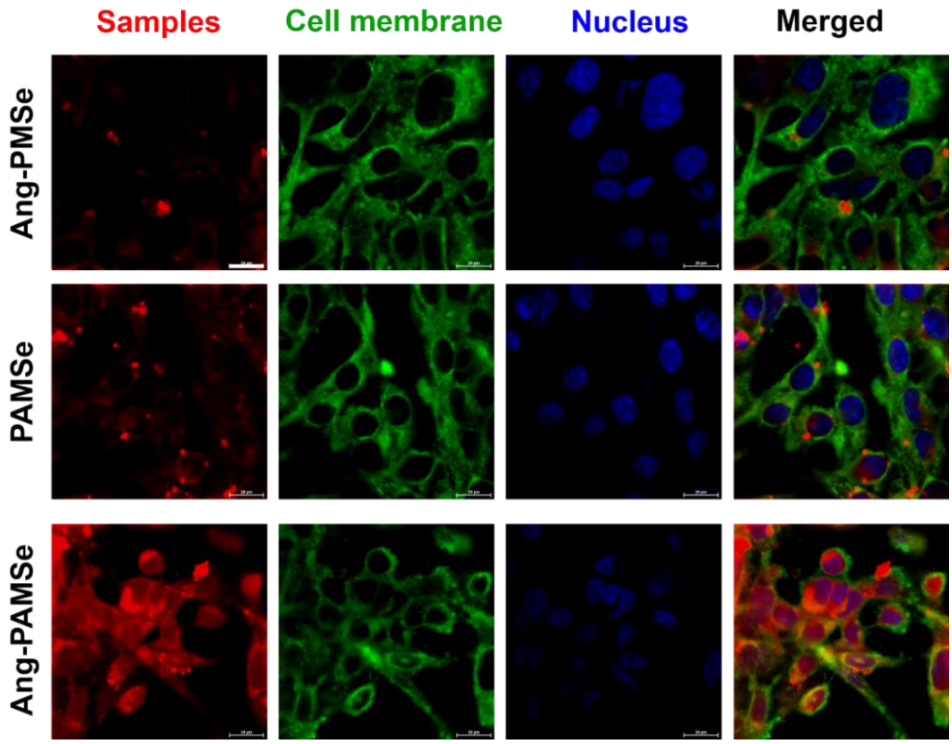

Supplementary Fig. 37 Representative CLSM images of GBM cells after different sample treatments for 24 h (Green, cell membranes stained with DiO; Red, samples were fluorescently labeled with cy5; Blue, cell nuclei stained with hochest33342; Scale bar: 20 μm, n=3 samples).

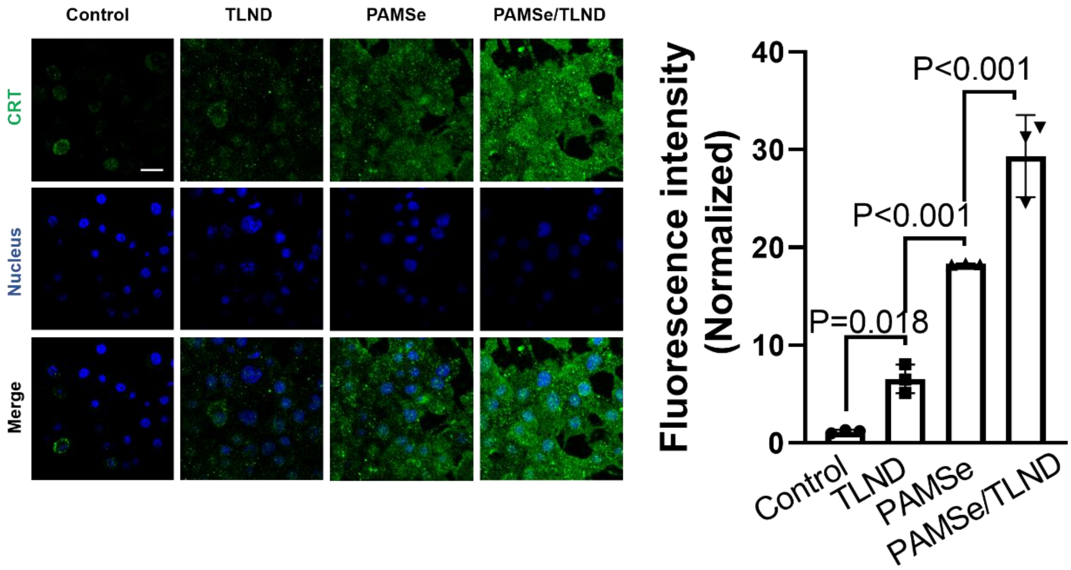

Supplementary Fig. 38 Representative CLSM images corresponding quantitative analysis of cell surface exposure of CRT (Green, CRT was immunofluorescently (FITC-488) labeled; Blue, cell nuclei stained with hochest33342; Scale bar: 20 μm). Data shown as the mean ± s.d. (n=3 independent experiments per group). Statistical significance was assessed by one-way ANOVA with post hoc LSD tests. Source data and exact p values are provided in the Source data file.

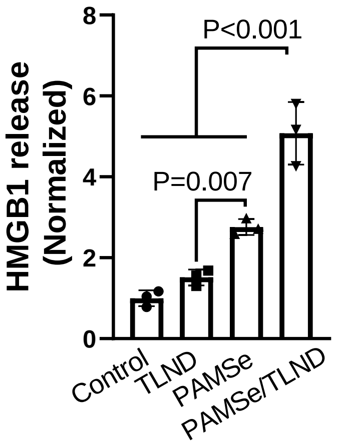

Supplementary Fig. 39 Quantitative analysis of extracellular release of high mobility group box 1 (HMGB1), and the fresh cell culture medium as control (n=3). Data shown as the mean ± s.d. (n=3 independent samples per group). Statistical significance was assessed by one-way ANOVA with post hoc LSD tests. Source data and exact p values are provided in the Source data file.

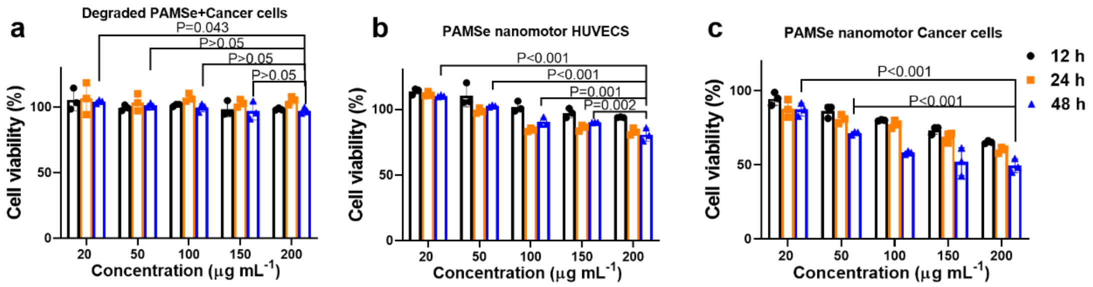

Supplementary Fig. 40 **a** The viability of cancer cells after treatment with degraded PAMSe (20, 50, 100, 150 and 200 μg mL^{-1}) for 48 h. **b** The viability of endothelial cells (HUVECs) after incubation with different concentrations of PAMSe nanomotors (20, 50, 100, 150 and 200 μg mL^{-1}) for 48 h. **c** The viability of cancer cells after incubation with different concentrations of PAMSe nanomotors (20, 50, 100, 150 and 200 μg mL^{-1}) for 48 h. Data shown as the mean ± s.d. (n=3 independent samples per group). Statistical significance was assessed by one-way ANOVA with post hoc LSD tests. Source data and exact p values are provided in the Source data file.

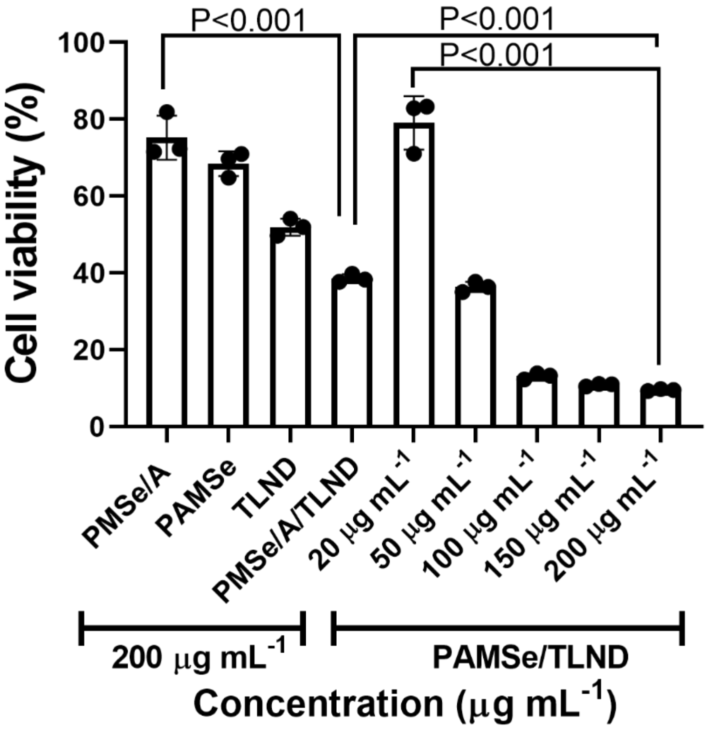

Supplementary Fig. 41 Cellular viability of GL261 cells after 24 h of sample treatment. The samples are PMSe/A, PAMSe, TLND and PMSe/A/TLND with concentration of 200 μg mL^{-1} and PAMSe/TLND with concentration of 20 μg mL^{-1}, 50 μg mL^{-1}, 100 μg mL^{-1}, 150 μg mL^{-1} and 200 μg mL^{-1} respectively. Data shown as the mean ± s.d. (n=3 independent samples per group). Statistical significance was assessed by one-way ANOVA with post hoc LSD tests. Source data and exact p values are provided in the Source data file.

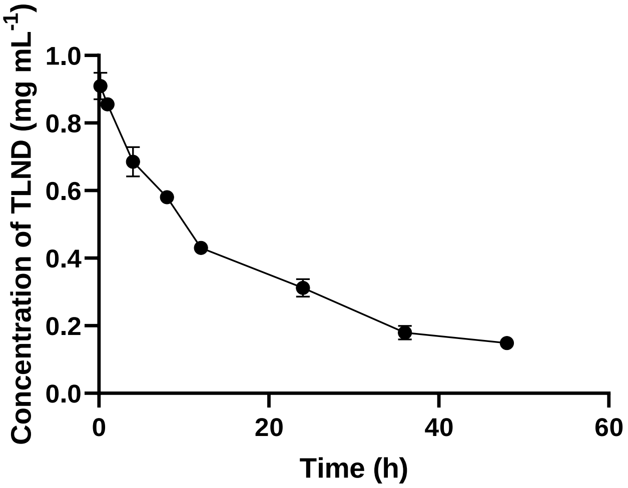

Supplementary Fig. 42 In vivo pharmacokinetics of Ang-PAMSe/TLND nanomotor in healthy SD rats. Experimental data are shown as mean ± s.d. of samples in a representative experiment. Data shown as the mean ± s.d. (n=3 independent samples per group). Source data are provided as a Source Data file.

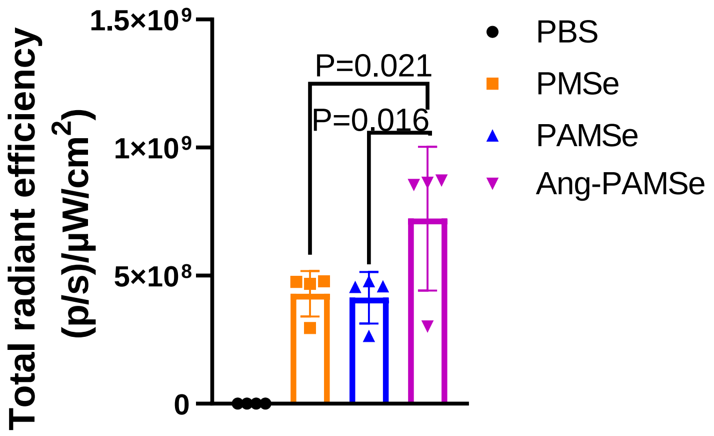

Supplementary Fig. 43 Total radiant efficiency of different cy5-labled samples PBS, Ang-PMSe, PAMSe, Ang-PAMSe in GBM. Data shown as the mean ± s.d. (n=4 independent samples per group). Statistical significance was assessed by one-way ANOVA with post hoc LSD tests. Source data are provided as a Source Data file.

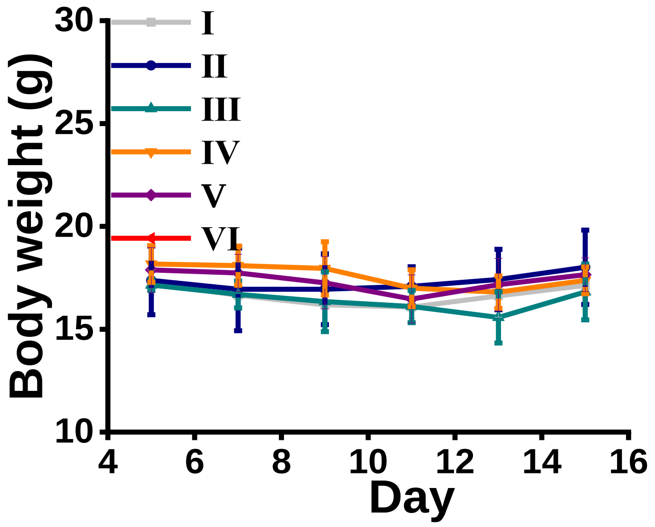

Supplementary Fig. 44 Body weight changes of mice during treatment process. The names corresponding to the samples are (I) Sham, (II) PBS, (III) TLND, (IV) Ang-PMSe/TLND, (V)) Ang-PAMSe and (VI) Ang-PAMSe/TLND). Data shown as the mean ± s.d. (n=3 independent samples per group). Source data are provided as a Source Data file.

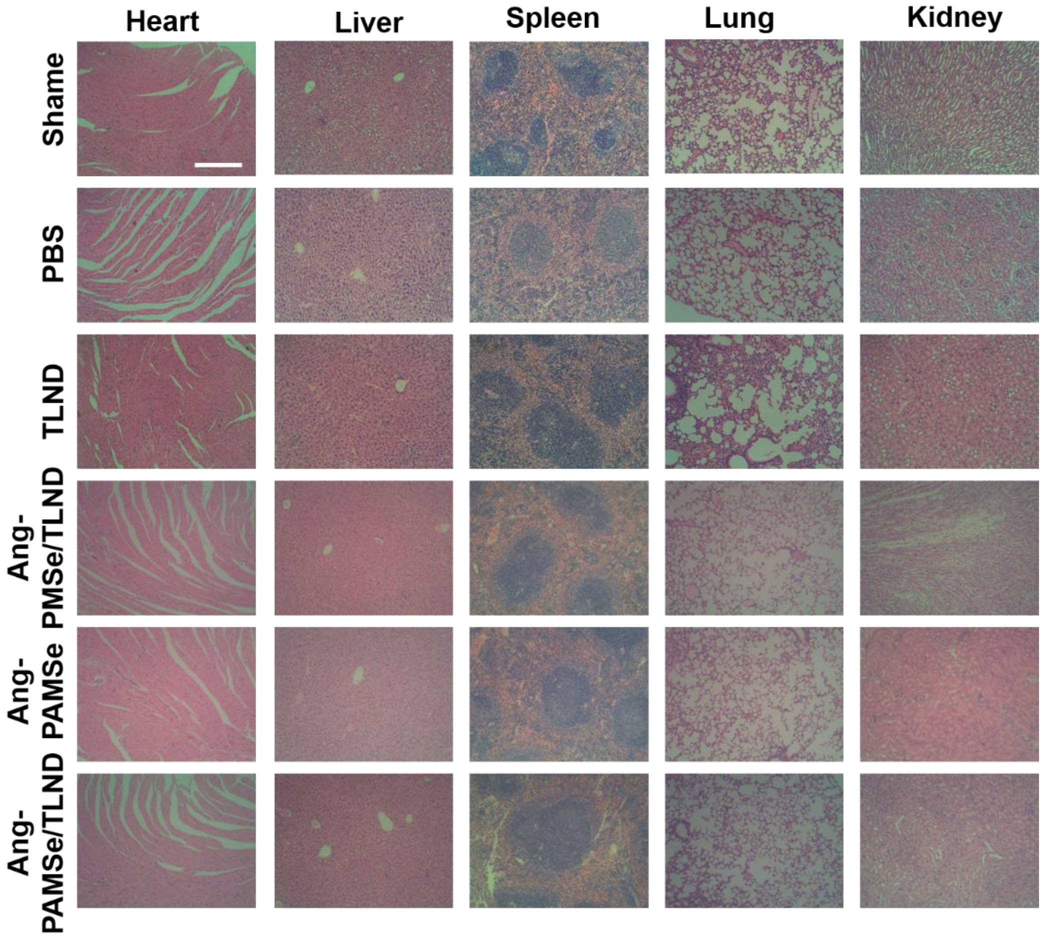

Supplementary Fig. 45 Histological analyses of different main organs (heart, liver, spleen, lung, and kidney) collected from orthotopic GL261 glioblastoma tumor-bearing C57 mice following different samples treatment (Scale bar: 500 µm, n=3 independent samples per group).

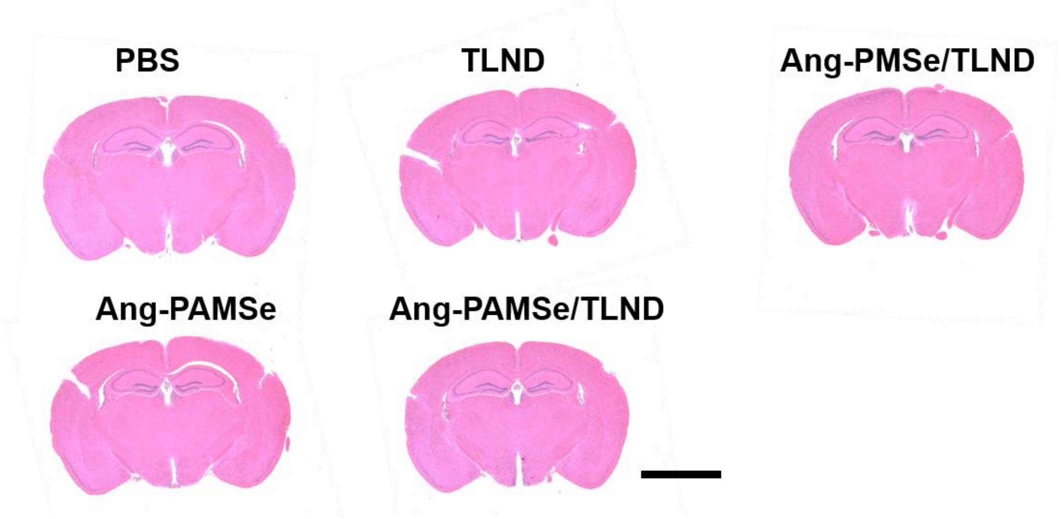

Supplementary Fig. 46 Histological observation of cerebral cortex tissue collected from healthy C57BL/6J mice after treated with PBS, TLND, Ang-PMSe/TLND, Ang-PAMSe and Ang-PAMSe/TLND (Scale bar: 3 mm, n=3 independent samples per group).

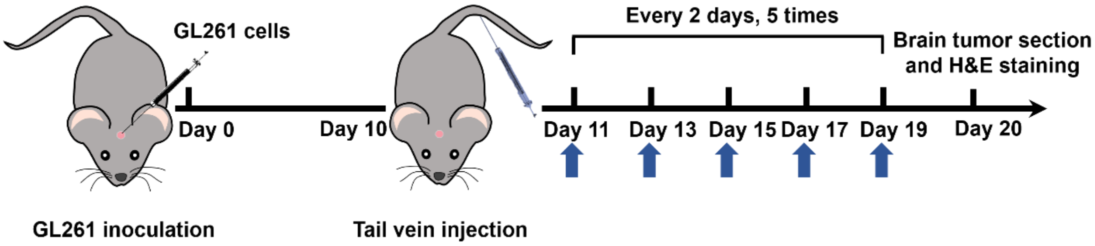

Supplementary Fig. 47 Schematic illustration of the orthotopic GL261 glioblastoma tumor-bearing C57BL/6J mice experimental design.

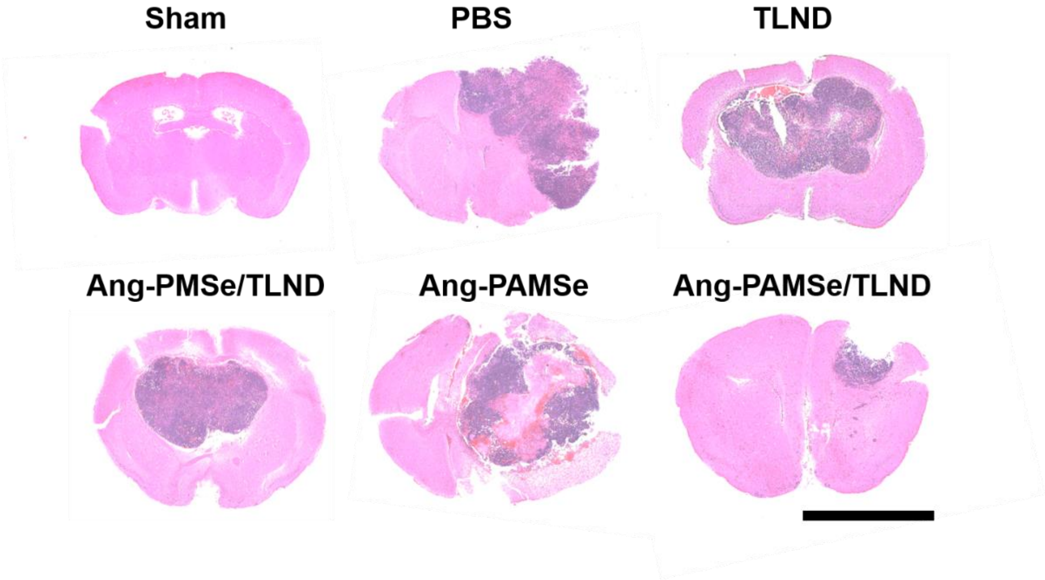

Supplementary Fig. 48 Histological analyses of brain tumor collected from orthotopic GL261 glioblastoma tumor-bearing C57BL/6J mice following different samples treatment (Scale bar: 4 mm) (n= 3 biologically independent animals per group).

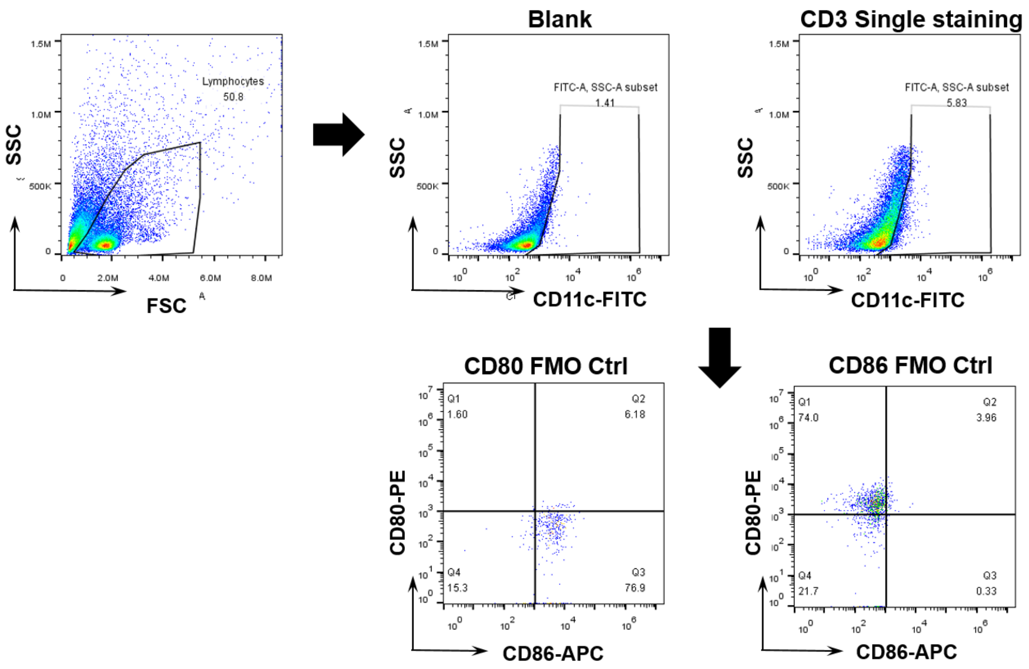

Supplementary Fig. 49 Gating strategy for CD80$^+$CD86$^+$ matured DCs analysis. The glioma tumor bearing mice from each group were euthanized on day 15. Tumor-draining lymph nodes tissue were harvested and the dissociated cells were collected for flow cytometry analysis to examine the DCs. Cells of interest were chosen based on size in the FSC/SSC plots, and then total lymphocyte cell number was determined by CD11c$^+$ cell counts, CD80$^+$ and CD86$^+$ lymphocyte cells were then gated within CD11c$^+$ lymphocytes, which was presented on Fig. 7a,b.

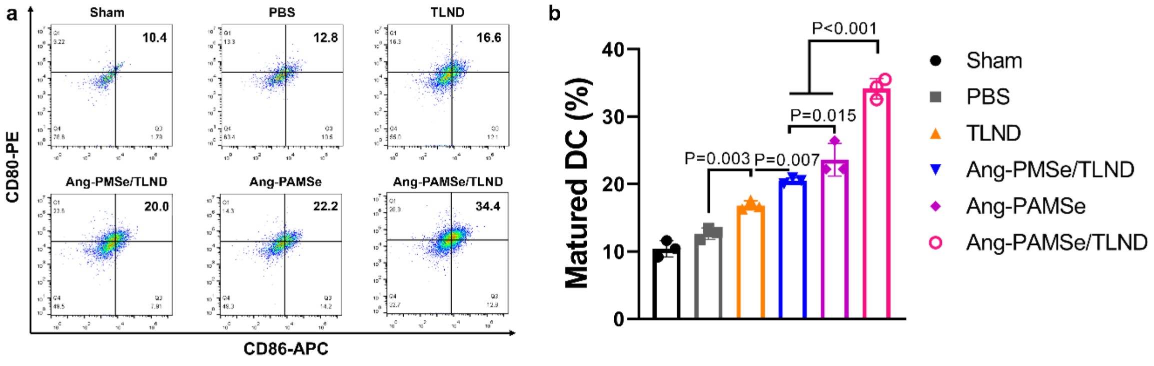

Supplementary Fig. 50 a Flow cytometry analysis of DCs maturation with each formulation, and **b** Frequency of DCs activation from the tumor tissue. Data shown as the mean ± s.d. (n=3 biologically independent experiments per group). Statistical significance was assessed by one-way ANOVA with post hoc LSD tests. Source data and exact p values are provided in the Source data file.

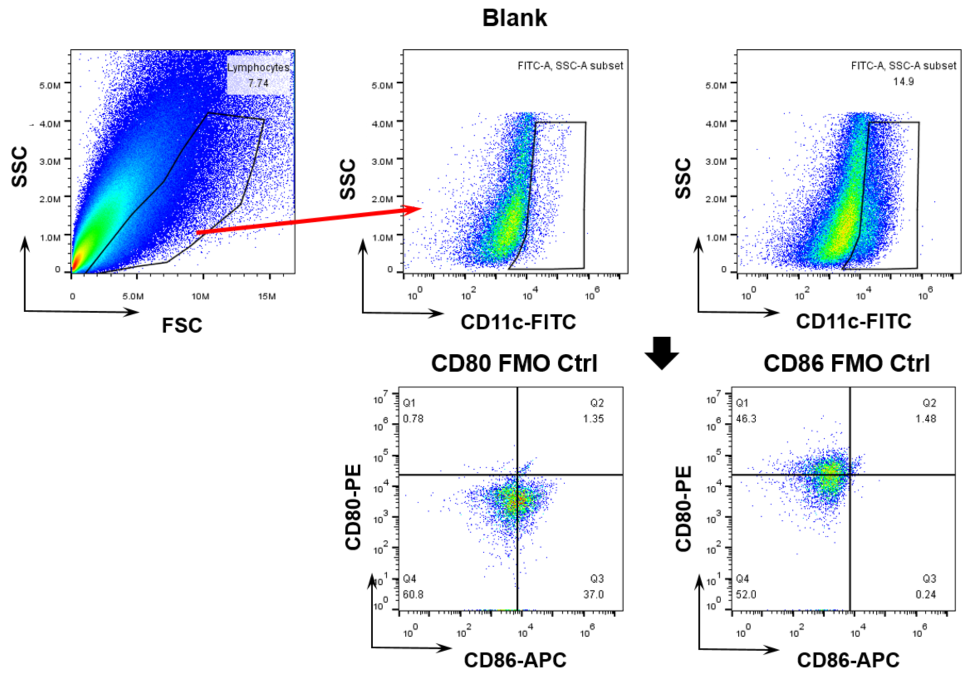

Supplementary Fig. 51 Gating strategy for CD80$^+$CD86$^+$ matured DCs analysis. The glioma tumor bearing mice from each group were euthanized on day 15. The perfused brain tissues were digested and the dissociated cells were collected for flow cytometry analysis to examine the DCs. Cells of interest were chosen based on size in the FSC/SSC plots, and then total lymphocyte cell number was determined by CD11c$^+$ cell counts, CD80$^+$ and CD86$^+$ lymphocyte cells were then gated within CD11c$^+$ lymphocytes, which was presented on Supplementary Fig. 50.

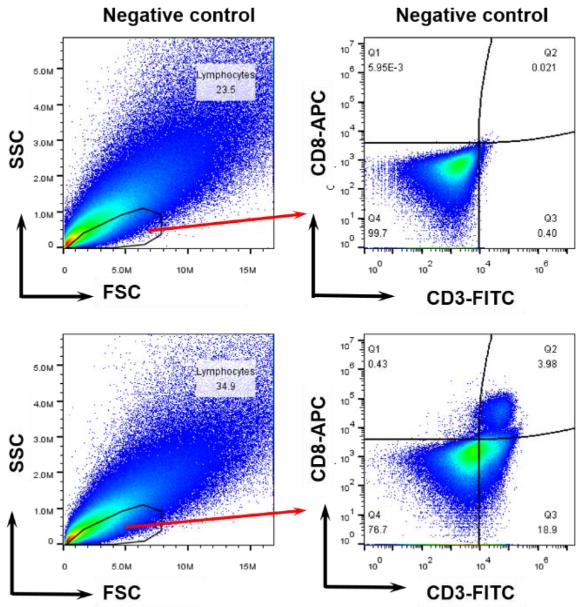

Supplementary Fig. 52 Gating strategy for CD3$^+$CD8$^+$ T cells analysis orthotopic GBM. The perfused brain tissues were digested and the dissociated cells were collected for flow cytometry analysis to examine the T lymphocytes. 100,000 events were collected for each sample in the analysis. Cells of interest were chosen based on size in the FSC/SSC plots, and then total T cell number was determined by CD3$^+$ cell counts and CD8$^+$ T cells were then gated within CD3$^+$ T lymphocytes, which was presented on Fig. 7c,d.

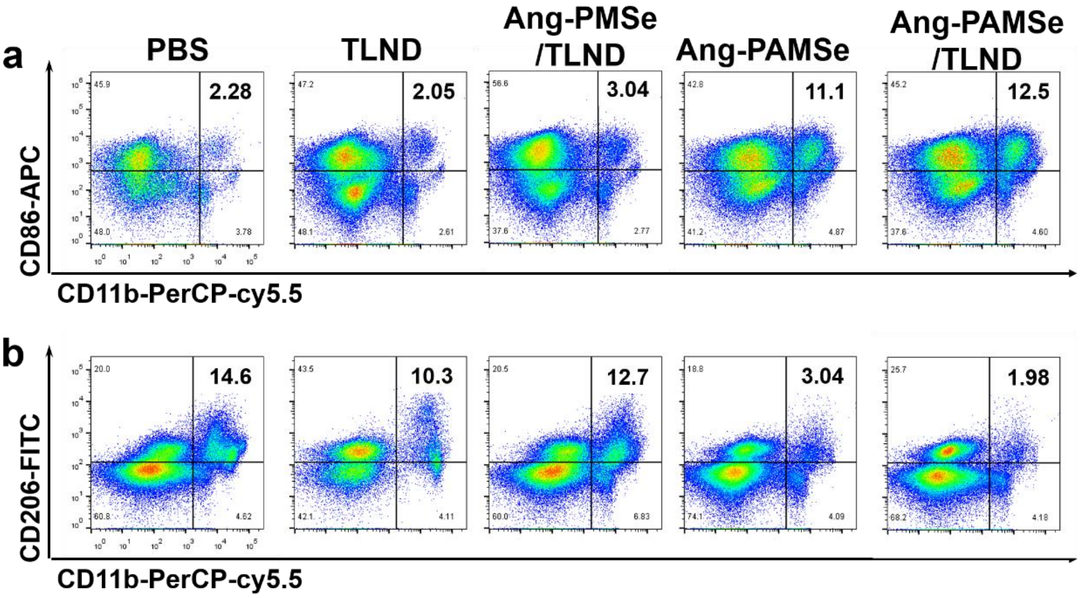

Supplementary Fig. 53 Representative flow cytometric plots of (**a**) M1-like macrophages ($CD11b^+CD86^+$) and (**b**) M2-like macrophages ($CD11b^+CD206^+$) in glioma-bearing brain tissue (n=3 biologically independent experiments per group).

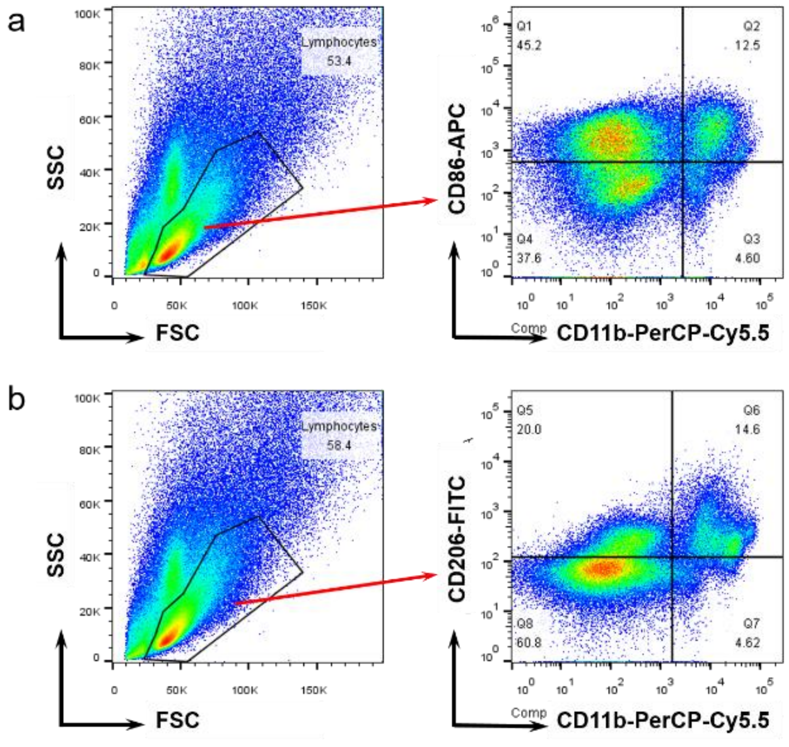

Supplementary Fig. 54 Gating strategy for macrophages cells analysis. The perfused brain tissues were digested and the dissociated cells were collected for flow cytometry analysis to examine the lymphocytes. 100,000 events were collected for each sample in the analysis. Cells of interest were chosen based on size in the FSC/SSC plots, and then total lymphocytes cell number was determined by $CD11b^+$ cell counts, (**a**) $CD86^+$ lymphocytes cells or (**b**) $CD206^+$ were then gated within $CD11b^+$ lymphocytes, which was presented on Fig.7e,f and Supplementary Fig. 53.

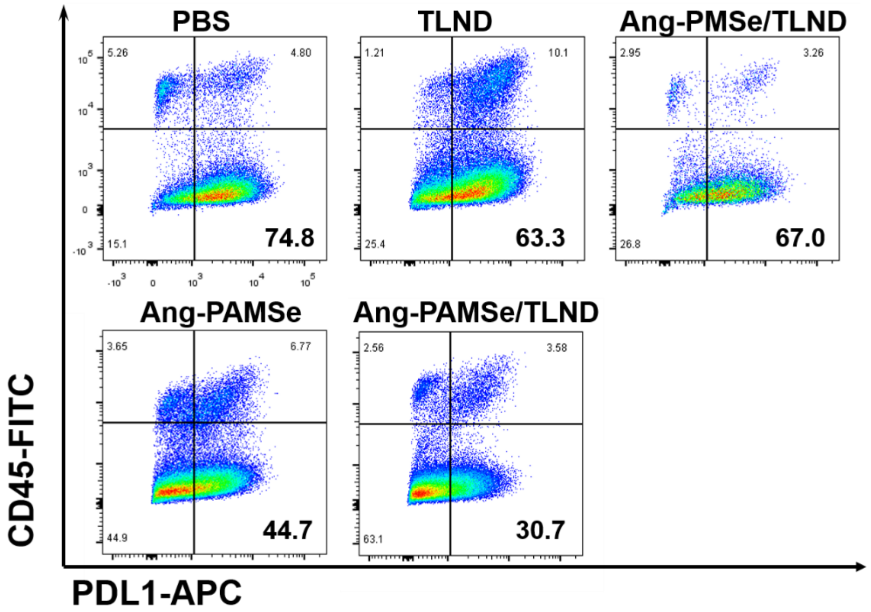

Supplementary Fig. 55 Representative flow cytometric plots of CD45^{-}PDL1^{+} glioma cells in glioma-bearing brain tissue (n=3 biologically independent experiments per group).

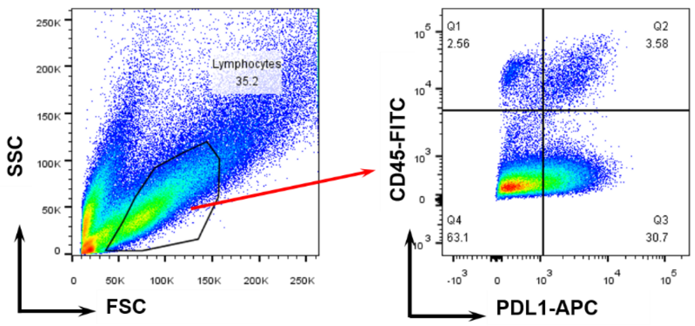

Supplementary Fig. 56 Gating strategy analysis for expression of PDL1 in tumor cells. The perfused brain tissues were digested and the dissociated cells were collected for flow cytometry analysis to examine the lymphocytes. 100,000 events were collected for each sample in the analysis. Cells of interest were chosen based on size in the FSC/SSC plots, and then total cell number was determined by CD45^{-} and PDL1^{+}, which was presented on Fig.7g and Supplementary Fig. 55.

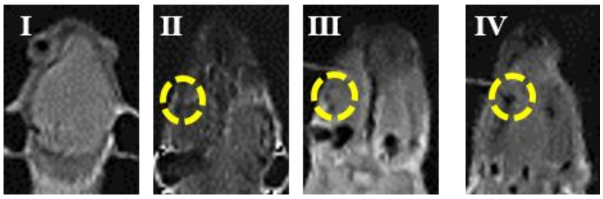

Supplementary Fig. 57 The T_2-weighted MRI and bioluminescence imaging of the sham-operated group and GBM mice (Yellow circles indicate MRI imaging (I) PBS, (II) TLND, (III) Ang-PMSe/TLND (IV) Ang-PAMSe, and (V) Ang-PAMSe/TLND) (n=3 biologically independent experiments per group).

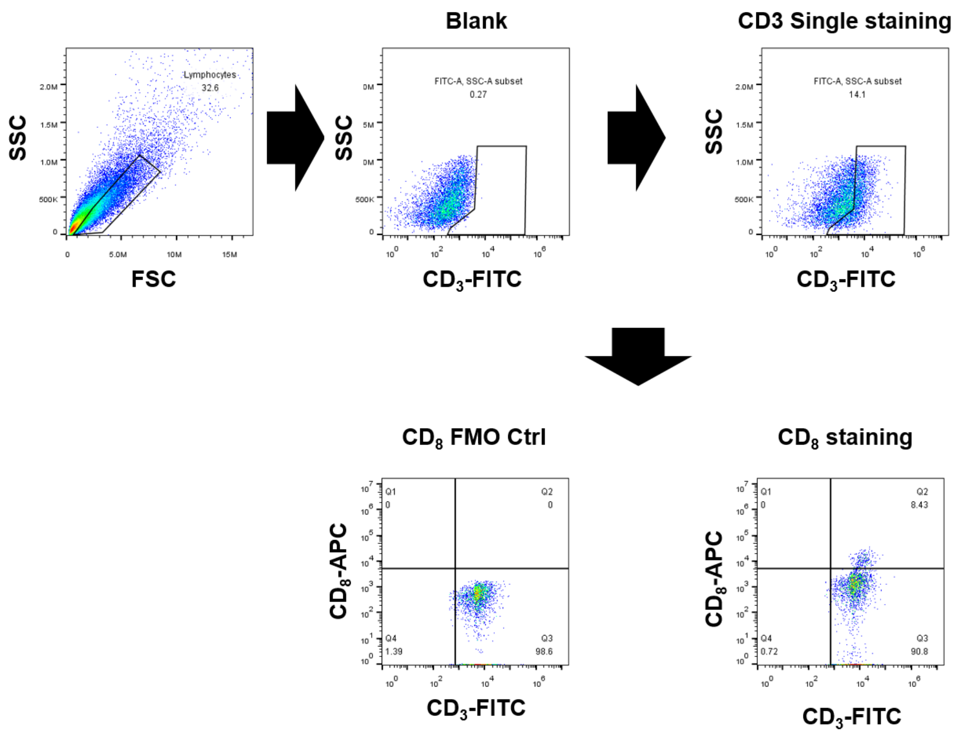

Supplementary Fig. 58 Gating strategy for $CD3^{+}CD8^{+}$ T cells analysis in distal brain tumors. The perfused brain tissues were digested and the dissociated cells were collected for flow cytometry analysis to examine the T lymphocytes. 100,000 events were collected for each sample in the analysis. Cells of interest were chosen based on size in the FSC/SSC plots, and then total T cell number was determined by $CD3^{+}$ cell counts and CD8+ T cells were then gated within $CD3^{+}$ T lymphocytes, which was presented on Fig. 8c,d.

Supplementary table 1. Summary of immune-related brain tumor treatments.

Samples	Immunomodulatory processes							Ref
	①	②	③	④	⑤	⑥	⑦	
DOX/R837/ALG OXA/R837/ALG	√ DOX/OXA	×	√ R837	×	√ R837	√ anti-PDL1	√ DOX /OXA	Sci. Adv. 2020[1]
THINR-CXCL10@Gel	√ MIT	×	√ siIDO1	×	√ CXCL10	×	×	Nat. Nanotechnol. 2021[2]
mAb nanocapsule	×	×	×	×	×	×	√ mAb	Adv. Mater. 2019[3
Epi/m+aPD1	√ Epi/m	√ Epi/m	×	×	√ Epi/m	√ aPD1	×	ACS Nano 2020[4]
VEGFR/Ang-2	×	×	×	√ cediranib MEDI3617	√ cediranib MEDI3617	×	×	P. Natl. Acad. Sci. USA 2015[5]
CPG ODN-loaded NPs	√ CpG ODN	×	√ CpG ODN	×	×	√ DMNP	√ DMNP	Nano Lett. 2020[6]
f(SLN)-iRGD:siRNA	×	×	×	×	√ siEGFR	√ siPDL1	×	ACS Nano 2019[7]
NIC	×	×	√ a-CTLA-4/a-PD-1	×	√ a-CTLA-4/a-PD-1	√ a-CTLA-4/a-PD-1	√ a-CTLA-4/a-PD-1	Nat. Commun. 2019[8]
iRGD-loaded SPNPs	×	×	√ STAT3i	×	√ STAT3i	×	√ STAT3i	Nat.Commun. 2020[9]
Ang-PAMSe/TLND	√ NO	√ NO	√ NO	√ NO	√ NO	√ NO	√ TLND	This work

① Promotes the release of tumor antigens
② Promotes tumor antigen presentation
③ T cell mobilization and activation
④ Enhanced T-cell transport
⑤ Enhanced T-cell infiltration
⑥ Enhancement of T-cell recognition of tumor cells
⑦ Killing tumor cells

Supplementary References

1. Chao, Y. et al. Localized cocktail chemoimmunotherapy after in situ gelation to trigger robust systemic antitumor immune responses. *Sci. Adv.* **6**, eaaz4204 (2020).
2. Zhang, J. et al. Immunostimulant hydrogel for the inhibition of malignant glioma relapse post-resection. *Nat. Nanotechnol.* **16**, 538-548 (2021).
3. Han, L. et al. Systemic delivery of monoclonal antibodies to the central nervous system for brain tumor therapy. *Adv. Mater.* **31**, 1805697 (2019).
4. Kinoh, H. et al. Translational Nanomedicine boosts anti-PD1 therapy to eradicate orthotopic PTEN-negative glioblastoma. *ACS Nano* **14**, 10127-10140 (2020).
5. Peterson, T. E. et al. Dual inhibition of Ang-2 and VEGF receptors normalizes tumor vasculature and prolongs survival in glioblastoma by altering macrophages. *P. Natl. Acad. Sci. USA* **113**, 4470-4475 (2016).
6. Ma, J. N. et al. Copresentation of tumor antigens and costimulatory molecules via biomimetic nanoparticles for effective cancer immunotherapy. *Nano Lett.* **20**, 4084-4094 (2020).
7. Erel-Akbaba, G. et al. Radiation-induced targeted nanoparticle-based gene delivery for brain tumor therapy. *ACS Nano* **13**, 4028-4040 (2019).
8. Galstyan, A. et al. Blood-brain barrier permeable nano immunoconjugates induce local immune responses for glioma therapy. *Nat. Commun.* **10**, 3850 (2019).
9. Gregory, J. V. et al. Systemic brain tumor delivery of synthetic protein nanoparticles for glioblastoma therapy. *Nat. Commun.* **11**, 5687 (2020).
