# Supplementary material for: A nitric-oxide driven chemotactic nanomotor for enhanced immunotherapy of glioblastoma
Source: Nat Commun. 2023 Feb 20;14:941. doi: 10.1038/s41467-022-35709-0 (PMC9941476; doi:10.1038/s41467-022-35709-0)
Supplement: Supplementary file 3 — Description of Additional Supplementary Files [file 41467_2022_35709_MOESM3_ESM.pdf]

Description of Additional Supplementary Files

Supplementary Movie 1: The trajectory tracking of PAMSe nanomotors under cellular condition.

Supplementary Movie 2: The trajectory tracking of PMSe/A nanomotors under cellular condition.

Supplementary Movie 3: The trajectory tracking of PAMSe and PMSe/A nanomotors in DMEM solution.

Supplementary Movie 4: Real-time live cell images of cancer cells treated with different type nanomotors.

Supplementary Movie 5: Chemotactic motion of samples in the Y model (Video is played at 5× speed).

Supplementary Movie 6: Chemotactic motion of samples in the microfluidic devices.
