# Supplementary material for: A nitric-oxide driven chemotactic nanomotor for enhanced immunotherapy of glioblastoma
Source: Nat Commun. 2023 Feb 20;14:941. doi: 10.1038/s41467-022-35709-0 (PMC9941476; doi:10.1038/s41467-022-35709-0)
Supplement: Supplementary file 10 — Reporting Summary [file 41467_2022_35709_MOESM10_ESM.pdf]

nature portfolio

Corresponding author(s): Chun Mao

Last updated by author(s): Dec 9, 2022

Reporting Summary

Nature Portfolio wishes to improve the reproducibility of the work that we publish. This form provides structure for consistency and transparency in reporting. For further information on Nature Portfolio policies, see our Editorial Policies and the Editorial Policy Checklist.

Statistics

For all statistical analyses, confirm that the following items are present in the figure legend, table legend, main text, or Methods section.

n/a	Confirmed	
☐	☒	The exact sample size (*n*) for each experimental group/condition, given as a discrete number and unit of measurement
☐	☒	A statement on whether measurements were taken from distinct samples or whether the same sample was measured repeatedly
☐	☒	The statistical test(s) used AND whether they are one- or two-sided<br>*Only common tests should be described solely by name; describe more complex techniques in the Methods section.*
☒	☐	A description of all covariates tested
☒	☐	A description of any assumptions or corrections, such as tests of normality and adjustment for multiple comparisons
☐	☒	A full description of the statistical parameters including central tendency (e.g. means) or other basic estimates (e.g. regression coefficient) AND variation (e.g. standard deviation) or associated estimates of uncertainty (e.g. confidence intervals)
☐	☒	For null hypothesis testing, the test statistic (e.g. *F*, *t*, *r*) with confidence intervals, effect sizes, degrees of freedom and *P* value noted<br>*Give P values as exact values whenever suitable.*
☒	☐	For Bayesian analysis, information on the choice of priors and Markov chain Monte Carlo settings
☒	☐	For hierarchical and complex designs, identification of the appropriate level for tests and full reporting of outcomes
☒	☐	Estimates of effect sizes (e.g. Cohen's *d*, Pearson's *r*), indicating how they were calculated

Our web collection on statistics for biologists contains articles on many of the points above.

Software and code

Policy information about availability of computer code

Data collection	Data were collected using JEOL JEM-2100F, Malvern Zetasizer Nano-Z, Bruker Avance 400 spectrometer, Liquid chromatography-mass spectrometry (6460 QQQ MS) with liquid chromatography (Agilent 1290 Infinity LC), Cary 5000 FTIR spectropho-tometer (Varian, USA), PerkinElmer 650 spectrophotometer (PerkinElmer Ltd., US), F-4600 spectrophotometer (Hitachi, Japan), gel permeation chromatography (GPC, Agilent 1260 Infinity Ⅱ), microplate reader (Multiskan FC, Thermo Fisher instruments Co., Ltd., US), fluorescence microscopy (MF53-N, Guangzhou Micro-shot Technology Co., Ltd., Guangzhou, China), confocal laser scanning microscopy (CLSM, HP Apo TIRF 100X N.A. 1.49, Nikon, Ti-E-A1R, Japan), small animal imager (IVIS Lumina III, Living image system), flow cytometry (BD Accuri C6 Plus flow cytometer).
Data analysis	Statistical analysis were performed using Microsoft Excel (2016), GraphPad Prism (Version 8), Origin 2018, ImageJ (Version 2.0.0), small animal imager (IVIS Lumina III, Living image system 4.5.5), CytExpert 2.3 and Flow Jo (Version 10), SPSS 13.0, ChemDraw (Ultra 7.0).

For manuscripts utilizing custom algorithms or software that are central to the research but not yet described in published literature, software must be made available to editors and reviewers. We strongly encourage code deposition in a community repository (e.g. GitHub). See the Nature Portfolio guidelines for submitting code & software for further information.

Data

Policy information about availability of data

All manuscripts must include a data availability statement. This statement should provide the following information, where applicable:

- Accession codes, unique identifiers, or web links for publicly available datasets
- A description of any restrictions on data availability
- For clinical datasets or third party data, please ensure that the statement adheres to our policy

All data that support the findings of this study are available within the article and the supplementary information. Source data are provided with this paper.

Human research participants

Policy information about studies involving human research participants and Sex and Gender in Research.

Reporting on sex and gender	N/A
Population characteristics	N/A
Recruitment	N/A
Ethics oversight	N/A

Note that full information on the approval of the study protocol must also be provided in the manuscript.

Field-specific reporting

Please select the one below that is the best fit for your research. If you are not sure, read the appropriate sections before making your selection.

[x] Life sciences [ ] Behavioural & social sciences [ ] Ecological, evolutionary & environmental sciences

For a reference copy of the document with all sections, see nature.com/documents/nr-reporting-summary-flat.pdf

Life sciences study design

All studies must disclose on these points even when the disclosure is negative.

Sample size	Sample sizes were determined to allow the statistical significance of differences of 50% or greater, and according to similar studies conducted in the field (Cao et al, Nature Communications 2019, 10:5783; Pan et al, Advanced Materials, 2021, 2007379). Details regarding the sample size of all experiments are provided in figure legends.
Data exclusions	No data was excluded.
Replication	All experiments were conducted at least three times and could be reliably reproduced. All experiments were performed as technical or biological replications as appropriate for the experiment design. Details of experimental replicates are given in the figure legends.
Randomization	The samples were divided into different groups randomly in all experiments.
Blinding	The investigators and authors have been consistently blinded to the group allocation during data collection and analysis.

Reporting for specific materials, systems and methods

We require information from authors about some types of materials, experimental systems and methods used in many studies. Here, indicate whether each material, system or method listed is relevant to your study. If you are not sure if a list item applies to your research, read the appropriate section before selecting a response.

Materials & experimental systems

n/a	Involved in the study
☐	☒ Antibodies
☐	☒ Eukaryotic cell lines
☒	☐ Palaeontology and archaeology
☐	☒ Animals and other organisms
☒	☐ Clinical data
☒	☐ Dual use research of concern

Methods

n/a	Involved in the study
☒	☐ ChIP-seq
☐	☒ Flow cytometry
☒	☐ MRI-based neuroimaging

Antibodies

Antibodies used

Primary antibodies:
Anti-calreticulin antibody (dilution 1:500, catalog number: 27298-1-AP, Proteintech)
Anti-CD31 antibody (dilution 1:100, catalog number: ab222783, clone: EPR17260-263, Abcam),
FITC-anti-mouse CD11c antibody (dilution 1:200, catalog number: 117305, clone: B277031, Lot: N418, Biolegend),
PE-anti-mouse CD80 antibody (dilution of 1:40, catalog number: 104707, clone: 16-10A1, Lot: B340153, Biolegend),
APC-anti-mouse CD86 antibody (dilution of 1:80, catalog number: 105071, clone: GL-1, Lot: B323580, Biolegend),
FITC-anti-mouse CD3 antibody (dilution of 1:50, catalog number: 100203, clone: 17A2, Lot: N418 Biolegend),
APC anti-mouse CD8 antibody (dilution of 1:80, catalog number: 100711, clone: 53-6.7, Lot: B329662, Biolegend),
Percp-cy5.5-anti-mouse CD11b antibody (dilution of 1:50, catalog number: 101228, clone: M1/70, Lot: B353753, Biolegend),
FITC-anti-mouse CD206 antibody (dilution of 1:100, catalog number: 141704, clone: C068C2, Lot: B350306, Biolegend),
FITC-anti-mouse CD45 antibody (dilution of 1:100, catalog number:103108, clone: 30-F11, Lot: B363202, Biolegend),
APC-anti-mouse PDL1 antibody (dilution of 1:50, catalog number: 124312, clone: 10F.9G2, Lot: B277024, Biolegend).

Secondary antibodies:
CoraLite488-conjugated Goat Anti-Rabbit IgG(H+L) (dilution of 1:50, Cat: SA00013-2, Clone: A0428, Lot: 20000127, Proteintech).

Validation

Antibodies used were commercially available and all antibodies were validated by manufacturers, with related data shown on the manufacturer website.
Primary antibodies:
Anti-calreticulin antibody (dilution 1:500, catalog number: 27298-1-AP, Proteintech)
https://www.ptgcn.com/products/Calreticulin-Antibody-27298-1-AP.htm
Anti-CD31 antibody (dilution 1:100, catalog number: ab222783, clone: EPR17260-263, Abcam),
https://www.abcam.cn/cd31-antibody-epr17260-263-bsa-and-azide-free-ab232533.html
FITC-anti-mouse CD11c antibody (dilution 1:200, catalog number: 117305, clone: B277031, Lot: N418, Biolegend),
https://www.biolegend.com/en-us/products/fitc-anti-mouse-cd11c-antibody-1815
PE-anti-mouse CD80 antibody (dilution of 1:40, catalog number: 104707, clone: 16-10A1, Lot: B340153, Biolegend),
https://www.biolegend.com/en-us/products/pe-anti-mouse-cd80-antibody-43
APC-anti-mouse CD86 antibody (dilution of 1:80, catalog number: 105071, clone: GL-1, Lot: B323580, Biolegend),
https://www.biolegend.com/en-us/products/apc-anti-mouse-cd86-antibody-2896
FITC-anti-mouse CD3 antibody (dilution of 1:50, catalog number: 100203, clone: 17A2, Lot: N418 Biolegend),
https://www.biolegend.com/en-us/products/fitc-anti-mouse-cd3-antibody-45
APC anti-mouse CD8 antibody (dilution of 1:80, catalog number: 100711, clone: 53-6.7, Lot: B329662, Biolegend),
https://www.biolegend.com/en-us/products/apc-anti-mouse-cd8a-antibody-150
Percp-cy5.5-anti-mouse CD11b antibody (dilution of 1:50, catalog number: 101228, clone: M1/70, Lot: B353753, Biolegend),
https://www.biolegend.com/en-us/products/percp-anti-mouse-human-cd11b-antibody-4315
FITC-anti-mouse CD206 antibody (dilution of 1:100, catalog number: 141704, clone: C068C2, Lot: B350306, Biolegend),
https://www.biolegend.com/en-us/products/fitc-anti-mouse-cd206-mmr-antibody-7318
FITC-anti-mouse CD45 antibody (dilution of 1:100, catalog number:103108, clone: 30-F11, Lot: B363202, Biolegend),
https://www.biolegend.com/en-us/products/fitc-anti-mouse-cd45-antibody-99
APC-anti-mouse PDL1 antibody (dilution of 1:50, catalog number: 124312, clone: 10F.9G2, Lot: B277024, Biolegend),
https://www.biolegend.com/en-us/products/apc-anti-mouse-cd274-b7-h1-pd-l1-antibody-6655

Secondary antibodies:
CoraLite488-conjugated Goat Anti-Rabbit IgG(H+L) (dilution of 1:50, Cat: SA00013-2, Clone: A0428, Lot: 20000127, Proteintech),
https://www.ptgcn.com/products/CoraLite488-conjugated-Affinipure-Goat-Anti-Rabbit-IgG-H-L-secondary-antibody.htm

Eukaryotic cell lines

Policy information about cell lines and Sex and Gender in Research

Cell line source(s)

The mouse GL261 (catalog: SAc0135)and GL261-Luc (catalog: IML-083)glioma cell lines were purchased from Shanghai

	Fusheng Industrial Co.; Mouse-derived brain endothelial cells (bEnd.3) (cell NO. CRL-2299) and human-derived glioma cells (U87) (cell NO. CL-0238) were purchased from Wuhan Procell Life Science&Technology Co., Ltd.; Human umbilical vein endothelial cells (HUVECs) were purchased from American type culture collection (ATCC, U.S., cell NO. CRL-1730); Michigan cancer foundation-7 cells (MCF-7, cell NO. 115) were obtained from Shanghai Meixuan Biotechnology Co., Ltd.
Authentication	None of the cell line used were authenticated.
Mycoplasma contamination	All cell lines tested negative for mycoplasma contamination.
Commonly misidentified lines (See ICLAC register)	None

Animals and other research organisms

Policy information about studies involving animals; ARRIVE guidelines recommended for reporting animal research, and Sex and Gender in Research

Laboratory animals	C57BL/6J mice (6-8 weeks, female) and Sprague-Dawley (SD, 6-8 weeks, female) rats were purchased from Jiangsu Alingfei Biotechnology Co. All animals were bred in a pathogen-free facility with a 12h light/dark cycle at 20±3 °C and 40-50% humidity and had ad libitum access to food and water.
Wild animals	No wild animals were used in the study.
Reporting on sex	No reporting on sex in the study.
Field-collected samples	No field collected samples were used in the study.
Ethics oversight	All animal experimental operations were in accordance with the specifications of the Guide for the Care and Use of Laboratory Animals, and all experimental procedures and protocols were approved by the Animal Experimentation Ethics Committee of Nanjing Normal University (approval number: IACUC-20200802-1).

Note that full information on the approval of the study protocol must also be provided in the manuscript.

Flow Cytometry

Plots

Confirm that:

- [x] The axis labels state the marker and fluorochrome used (e.g. CD4-FITC).
- [x] The axis scales are clearly visible. Include numbers along axes only for bottom left plot of group (a 'group' is an analysis of identical markers).
- [x] All plots are contour plots with outliers or pseudocolor plots.
- [x] A numerical value for number of cells or percentage (with statistics) is provided.

Methodology

Sample preparation	In situ tumour-bearing mice were taken at the end of the above treatment and euthanised. Mouse lymph nodes were taken, ground, and placed in staining buffer through a 70 µm pore size filter. The above cell suspension was centrifuged (1500 rpm, 5 min) and the supernatant was removed. Staining buffer was added to blow the cells well. First 10 µL of FC blocking (1 µg/106 cell) was added to each sample tube, and they were shaken and mixed well to avoid non-specific antibody binding. After incubation on ice for 30 min, the supernatant was discarded by centrifugation, and 100 µL of staining buffer was added for staining.<br>The perfused mouse brains were rinsed in Hank's buffer solution to remove the surface foreign matter, and the brain tissues were cut to the size of rice grains with surgical scissors and added with 1 mg mL-1 collagenase IV and 50 U mL-1 DNase I in 1640 medium in 5 mL of tissue digest, placed on a 37°C shaker, and fully digested for 1 h. The entire digested tissue homogenate was poured into a 70 µm nylon filter, and the tissue residue was filtered by grinding the tissue with the flat end of a syringe, the above cell suspension centrifuged to remove the supernatant. The cells were blown well by adding staining buffer.
Instrument	BD Accuri C6 Plus flow cytometer
Software	CytExpert 2.3 and Flow Jo (Version 10)
Cell population abundance	At least 10,000 relevant events were acquired for all FACS analysis.
Gating strategy	In general, cells were first gated on FSC/SSC. Singlet cells were gated using FSC-H and FSC-A. Dead cells were then excluded and further surface and intracellular antigen gating was performed on the live cell population.

- [x] Tick this box to confirm that a figure exemplifying the gating strategy is provided in the Supplementary Information.
